# Endogenous Site-Specific Encoding of Trifluoromethyl-Bearing Phenylalanine and Tryptophan for in-Cell ^19^F NMR

**DOI:** 10.1021/jacs.5c18349

**Published:** 2026-02-13

**Authors:** George Augustin, Fatema Bhinderwala, Nathan D. Alexander, Iker Hernández, Stanislau Stanisheuski, Christina M. Monnie, Alex J. Eddins, Yogesh M. Gangarde, Vadim A. Soloshonok, Mikel Oiarbide, Aitor Landa, Richard B. Cooley, Angela M. Gronenborn, Ryan A. Mehl

**Affiliations:** † Department of Biochemistry and Biophysics, 2011 Agricultural and Life Sciences, 2694 Oregon State University, Corvallis, Oregon 97331, United States; ‡ Department of Structural Biology, 12317 University of Pittsburgh School of Medicine, 3501 Fifth Ave., Pittsburgh, Pennsylvania 15261, United States; § Department of Organic Chemistry I, Faculty of Chemistry, University of the Basque Country UPV/EHU, Paseo Manuel Lardizabal 3, 20018 Donostia-San Sebastián, Spain; ∥ IKERBASQUE, Basque Foundation for Science, Bilbao 48011, Spain

## Abstract

Understanding protein structure, dynamics, and interactions in live mammalian cells is essential for elucidating cellular mechanisms in health and disease. Here, we report genetic code expansion (GCE) systems that enable efficient site-specific incorporation of trifluoromethylphenylalanine (tfmF) and trifluoromethyltryptophan (tfmW) into mammalian proteins. While tfmF has previously been encoded in *E. coli* for electroporation-based in-cell ^19^F NMR, we establish the first system for direct tfmF encoding in mammalian cells. Moreover, we developed entirely new GCE tools for tfmW, enabling its incorporation in both *E. coli* and mammalian cells, the first report of tfmW encoding for ^19^F NMR. Using these systems, we expressed fluorinated cyclophilin A in HEK293T cells, compared the sensitivity of the in-cell NMR spectra with those obtained by electroporation, and assessed cyclosporin A binding. This work establishes the first mammalian-cell expression of tfm-labeled proteins, expanding the toolkit of fluorine probes for in-cell NMR.

## Graphical abstract

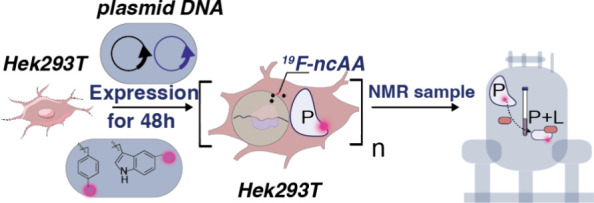

Our ability to investigate macromolecular structure and dynamics in living cells is critical for understanding cellular processes. In-cell NMR spectroscopy is an emerging method for studying protein dynamics, interactions, and conformations of biomolecules at the atomic level in mammalian cells.^1–3^ Traditional heteronuclear ^15^N, ^13^C in-cell NMR applications require isotopically enriched proteins, with spectra frequently adversely affected by cellular background signals or signal loss due to protein interactions with cellular components,^4,5^ manifested by severe line broadening. These challenges are mitigated by coupling genetic code expansion (GCE) with ^19^F NMR spectroscopy.^4^

^19^F is an ideal NMR probe for in-cell NMR studies, as it is 100% naturally abundant with exquisite chemical shift responsivity and high sensitivity to its environment. Thus, signal overlap is not an issue, and 1D spectroscopy suffices. Fluorine is also absent from mammalian cells, rendering any spectra background-free.^6,7^

Residue-specific encoding of monofluorinated amino acids has been extensively used over the past 20 years, as fluorine-substituted amino acids closely resemble native amino acids and are efficiently incorporated by endogenous aminoacyl-tRNA synthetases (RS) and tRNA (RS/tRNA) pairs.^8–13^ Although recently shown in human cells, this approach often causes heterogeneous labeling and proteome-wide ^19^F background.^14^ Incorporating trifluoromethyl-bearing noncanonical amino acids (tfm-ncAAs) offers advantages for in-cell NMR applications, given the higher intensity and smaller line widths of CF_3_ resonances compared to those of amino acids bearing a single F atom.^15,16^ Additionally, their structural difference from canonical amino acids reduces mischarging by natural RS/tRNA pairs, minimizing background encoding, and enables GCE-engineered site-specific encoding by RS/tRNA_UAG_.^17,18^

While bacterial GCE systems exist for tfmF, none are compatible with mammalian cell expression. Consequently, current tfmF-labeled proteins for in-cell NMR are typically produced in *E. coli* and delivered into mammalian cells by electroporation.^19^

To generate mammalian GCE encoding systems for trifluoromethyl-containing amino acids, we initially focused on evolving the mammalian compatible *Methanomethylophilus alvus* (*Ma*) PylRS/tRNA_CUA_ pair to incorporate 4-trifluoromethylphenylalanine (tfmF) and 5-trifluoromethyltryptophan (tfmW) in response to the amber codon, since this *Ma* pair has been successfully engineered to accommodate a wide range of structurally diverse aromatic ncAAs. TfmF is commercially available, and tfmW was synthesized using a strategy similar to our prior work on difluoro-Trp syntheses^20^ (Figure S1, and details of the synthesis and characterization are provided in SI). Starting with a 5-site *Ma*-PylRS library containing 3.2 million RS variants, standard positive, negative, and fluorescence-based selection strategies were employed for the selection, as previously described (Figure S2).^21,22^

Selection with tfmW at 400 μM yielded a single efficient tfmW aaRS protein variant, with three distinct codon sequences, all capable of site-specific tfmW encoding at site 150 of superfolder GFP (tfmW-sfGFP^150^), with efficiency matching wild-type sfGFP (sfGFP^WT^) in *E. coli* (Figure S3A-E). The tfmW concentration-dependent expression of tfmW-sfGFP^150^ was very similar for the three genetic variants and exceeded sfGFP^WT^ expression yields when cells were supplemented with >500 μM tfmW (Figure 1A). The A2 RS gene, designated here as tfmW-RS, was used for all downstream bacterial expression and mammalian encoding optimization (Figure S3A-E). Mass spectrometry (MS) was used to confirm the site-specific incorporation of tfmW into sfGFP (Figure S3F). To simplify tfmW-protein expression using pET plasmids in *E. coli*, tfmW-RS was cloned into the medium copy-number pAJE1 plasmid,^23^ containing a constitutively expressed *Ma* tRNA_CUA_.^22^ tfmW-sfGFP^150^ protein yields in the presence of 400 μM tfmW matched sfGFP^WT^ yields, and site-specific encoding was confirmed using MS (Figure S3F). This efficient bacterial tfmW system expands the site-specific ^19^F NMR toolbox and was adapted for mammalian use.

**Figure 1:**
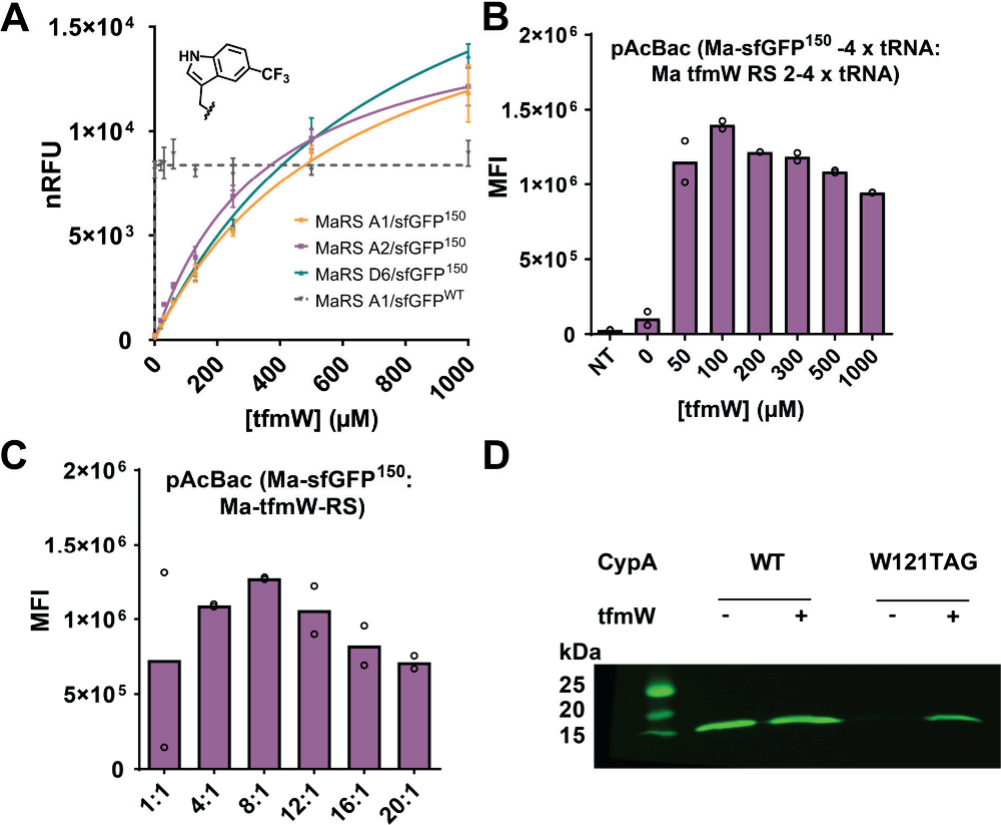
Expression of tfmW-proteins using a tfmW-RS/tRNA pair in *E. coli* and mammalian cells. (A) Expression of tfmW-sfGFP^150^ in *E. coli* with 0–1 mM tfmTrp, quantified by normalized relative fluorescence (nRFU). (B) Concentration-dependent expression of tfmW-sfGFP^150^ in HEK293T cells, quantified by the mean fluorescence intensity (MFI). (C) TfmW-sfGFP^150^ expression in HEK293T cells at different pAcBac1-Ma-sfGFP150:pAcBac1-tfmW-RS plasmid ratios. (D) tfmW-CypA^121^ HEK293T cell expression detected by Western blotting using anti-FLAG antibodies.

For mammalian incorporation, the tfmW-RS was cloned into a pAcBac1 plasmid containing 4 *Ma* tRNA_CUA_ genes (Figure S4A). Cotransfection of the resulting pAcBac1-tfmW-RS plasmid with the fluorescent reporter plasmid pAcBac1-sfGFP-TAG150, containing fourx tRNA_CUA_ genes in HEK293T cells, enabled sfGFP expression only upon addition of tfmW (Figure S4B). The optimal tfmW concentration (100 μM) for producing tfmW-sfGFP^150^ was determined by adding tfmW from 0 to 1 mM (Figure 1B). Consistent with previous work, an optimal pAcBac1 plasmid ratio of 8:1 (expression gene: RS) produced a maximum tfmW-protein expression at 100 μM tfmW (Figure 1C).^22^ Using the optimized tfmW-encoding conditions in HEK293T cells, we confirmed site-specific, high-fidelity encoding of tfmW by characterizing purified tfmW-sfGFP^150^ through whole protein MS analysis (Figure S4C).

Screening of the *Ma* PylRS/tRNA_CUA_ library did not yield an efficient RS/tRNA pair selective for tfmF. This was unexpected given previous success with similarly sized ncAAs such as acridonylalanine^22^ and tetrazine-substituted phenylalanine,^24^ although we did note challenges in generating *Ma* PylRS for small para-substituted phenylalanine ncAAs (unpublished results). To circumvent this issue, we evaluated an efficient, polyspecific *Ec*TyrRS/tRNA_CUA_ that was engineered to accept phenylalanine with a range of para-substituted functional groups and is orthogonal in mammalian cells.^25–30^

HEK293T cells were transfected with the pGA2 plasmid, which encodes the engineered *Ec*TyrRS driven by the mouse phosphoglycerate kinase 1 promoter (pGK), and four tandem copies of tRNA_CUA_, together with a pUC plasmid containing the sfGFP^150^ gene, controlled by an eukaryotic translational elongation factor 1 alpha (EF1α) promoter (Figure S5A). Cells were cultured with varying concentrations of tfmF and pAzF (Figure 2A), and the expression of ncAA-sfGFP was quantified by flow cytometry. We observed that incorporation of tfmF via the *Ec*TyrRS/tRNA pair occurred with approximately 50% efficiency for pAzF, reaching maximal suppression at 500 μM for both ncAAs. To test whether additional tRNAs improved efficiency, four tRNA repeats were added to the sfGFP plasmid, mimicking the tfmW-pAcBac1 design. Accordingly, the RS gene in pGA2 was replaced with the sfGFP^150^ gene under the EF1α promoter, yielding pGA1-GOI (Figure S5A, B).

**Figure 2:**
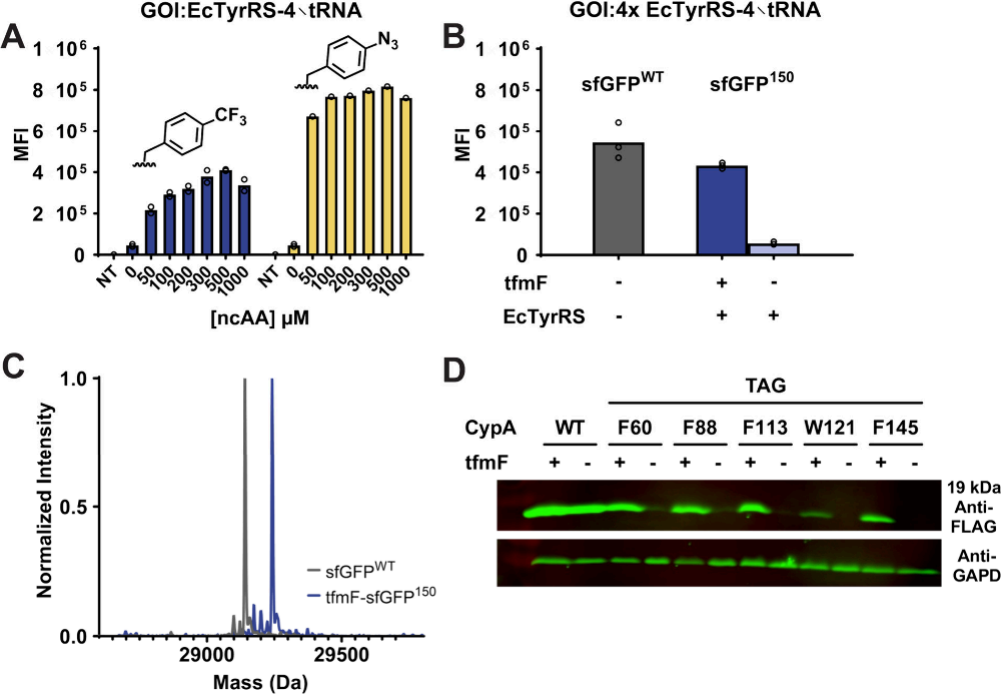
Expression of tfmF-sfGFP^150^ and tfmF-CypA in HEK293T cells. (A) Expression of tfmF-sfGFP^150^ and pAzF-sfGFP^150^ via *Ec*TyrRS/tRNA_CUA_ in HEK293T cells for 0–1.0 mM ncAAs. (B) Comparison of tfmF-sfGFP^150^ and sfGFP^WT^ expression at 500 μM tfmF (tfmF-sfGFP^150^ expression is 80% of the sfGFP^WT^ yield). (C) ESI-Q-TOF MS of tfmF-sfGFP^150^ (29242 obs; predicted 29242 Da) and sfGFP^WT^ (29141 obs; predicted 29142 Da). (D) tfmF-CypA expression in HEK293T detected by anti-FLAG Western blotting.

Maximal tfmF-sfGFP^150^ expression in HEK293T cells was achieved at a 1:1 ratio (Figure S5C) of transfected pGA1:pGA2 (GOI: GCE) plasmids. No further improvement was seen after the addition of extra tRNAs (Figure 2B). Thus, optimal expression of tfmF-sfGFP^150^ was obtained by using 500 μM tfmF with a 1:1 plasmid ratio, producing ∼80% of the sfGFP^WT^ levels (Figure 2B). High-fidelity encoding was confirmed by purifying tfmF-sfGFP^150^ from HEK293T cells and verifying its identity by MS (Figure 2C).

With high-fidelity, efficient mammalian GCE systems for tfmW and tfmF established, we next compared direct protein expression in HEK293T cells to the conventional electroporation of *E. coli*-expressed proteins.

As a proof of concept, we selected Cyclophilin A (CypA), a highly abundant cytosolic peptidyl-prolyl isomerase essential for protein folding and a key accessory factor in HIV-1 infectivity.^31,32^ CypA binds tightly to the immunosuppressant drug cyclosporine A (CsA), resulting in a well-characterized conformational change that inhibits T cell activation.^33^ Our prior ^19^F NMR work using *E. coli* GCE tfmF encoding systems^18^ established that tfmF labeling at position F60 is optimal for monitoring CsA ligand binding. Similarly, substitution of W121 with 5-fluorotryptophan (5FW) produces pronounced ^19^F chemical shift changes upon CsA binding.^34^ To assess the new GCE systems, we constructed mammalian-compatible CypA expression plasmids bearing C-terminal His tags (Figure S6).

The sfGFP gene in pAcBac1-GOI and pGA1-GOI plasmids was replaced with cypa, and TAG codons were introduced at positions encoding for F60 and W121. Efficient tfmW- and tfmF CypA incorporation was verified by western blotting (Figures 1D and 2D), and the identity of purified tfmF-CypA^60^ and tfmW-CypA^121^ from HEK293T cells was confirmed by mass spectrometry (Figure S6D). As expected, all purified proteins contained an N-terminal acetyl group (Figure S7).^35,36^

At this stage, all components required to compare tfmF and tfmW encoding for in-cell NMR were in hand. We expressed and purified tfmF-CypA^60^ and tfmW-CypA^121^ using the two optimized *E. coli* expression systems (Figures S8 and S9). The ^19^F spectra of these proteins in buffer, recorded with and without CsA (Figure 3A), showed the expected (+0.8 ppm) upfield shift for tfmF-CypA^60^ upon CsA binding, consistent with previous reports.^4^

**Figure 3:**
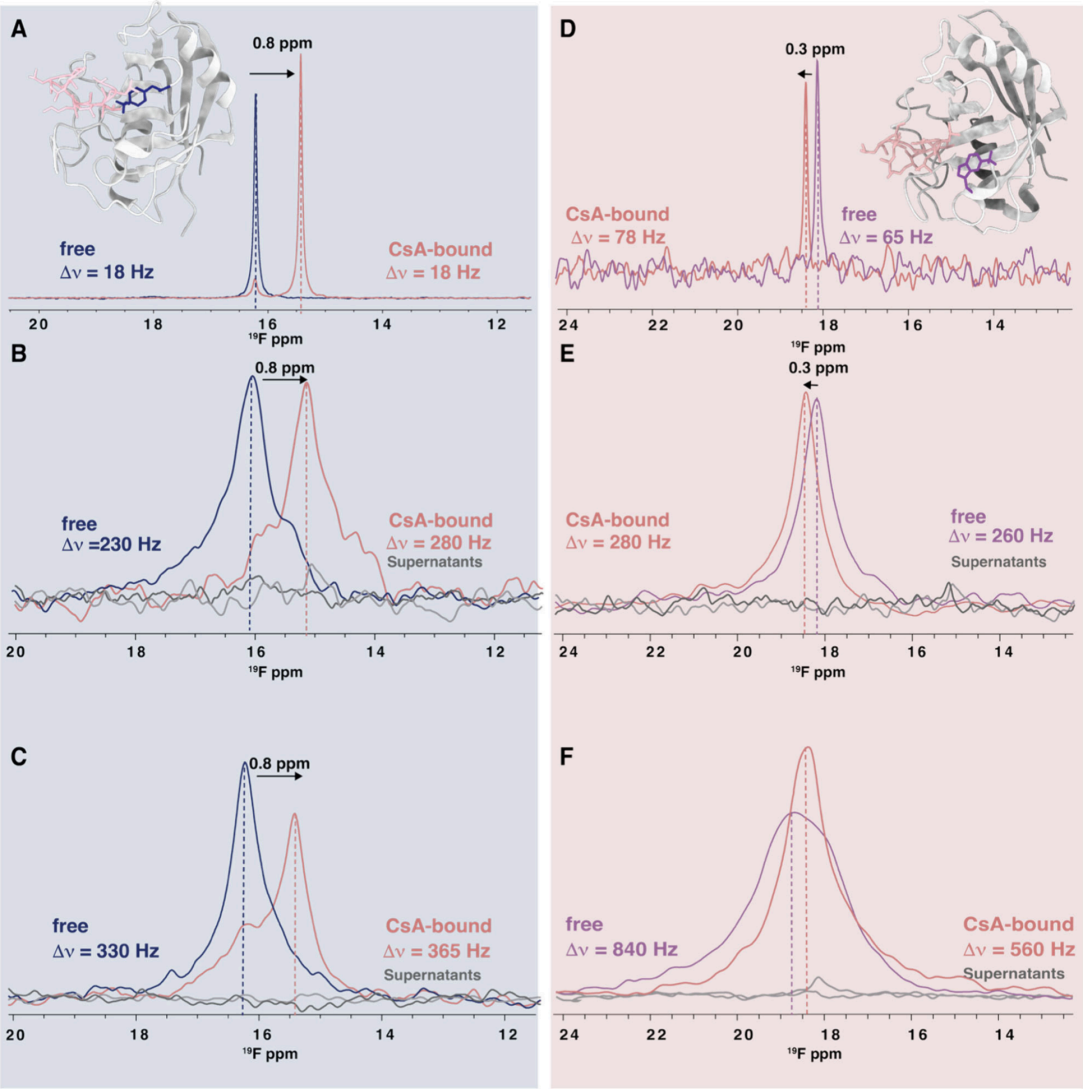
In-cell ^19^F NMR spectra of tfmF- and tfmW-labeled CypA. Spectra of tfmF-CypA^60^ without and with CsA are shown in blue and pink, respectively, (A) in buffer, (B) in cells after electroporation, or (C) following transient transfection. ^19^F spectra of tfmW-CypA^121^ without and with CsA are shown in purple and pink, respectively, (D) in buffer, (E) in cells after electroporation, and (F) following transient transfection. Supernatant control spectra, recorded after the in-cell spectra, are shown in gray. Structures of the CypA/CsA complex (PDB ID 1CWA) are inset in panels A and D, with CypA in gray ribbon representation, CsA in pink stick representation, and tfm-ncAA side chains in purple stick representation, respectively. The sample contained 56 million cells, electroporation was carried out using 3 mM CypA as the outside protein solution, and cells were treated with 50 μM CsA for 60 minutes before measuring the spectra of the CypA/CsA complex.

We also compared in-cell spectra obtained after transient transfection with the GCE machinery to those from the electroporation of HEK293T with *E. coli*-expressed tfmF-CypA^60^, as electroporation-based in-cell NMR presently is the standard practice. For this comparison, tfmF-CypA^60^ was expressed and purified from *E. coli* and subsequently delivered into HEK293T cells by electroporation.

Using 56 million HEK293T cells, ^19^F 1D spectra were recorded for 60 min, with the signal readily detected within the first 10 minutes (Figure 3B). After the initial measurements of tfmF-CypA^60^-containing cells, the cells were treated with 50 μM CsA for 60 minutes. Subsequently recorded 1D spectra showed the disappearance of the uncomplexed tfmF-CypA^60^ resonance at 16.0 ppm (blue), and the CsA-bound CypA resonance appearing at 15.5 ppm (pink, Figure 3B). The ^19^F frequencies for the uncomplexed and CsA-bound tfmF-CypA^60^ are identical to those observed in buffer (Figure 3A) and A2780 cells.^37^ The line width of the CsA-bound ^19^F resonance was slightly broader (280 Hz) than that of the uncomplexed CypA (230 Hz), in contrast to our observation in A2780 cells, where the ^19^F line width of the CypA-CsA complex resonance was smaller than that of tfmF-CypA^60^ alone. This finding agrees with results from our previous work where incorporation of tfmF at the F60 position reduces CsA affinity from 10 to 30 nM to approximately 7.0 ± 0.4 μM for tfmF60 CypA/CsA, yielding a *K*_d_ value about 200-fold higher than that reported for wildtype CypA/CsA.^19^

Next, HEK293T cells were transfected with plasmids encoding the GCE machinery and CypA^F60TAG^ expressing tfmF-CypA^60^ for 48 h to achieve protein levels comparable to those of electroporated samples. The resulting ^19^F spectra (Figure 3C) resembled those after electroporation, although resonances were broader. Whether this is due to specific or nonspecific interactions of N-acetylated protein with cellular components requires further investigation.

We compared the tfmW-CypA^121^ expression and ^19^F spectra in buffer (Figure 3D) and after electroporation (Figure 3E). In the buffer, tfmW-CypA^121^ resulted in a sharp 65 Hz resonance that shifted downfield by 0.3 ppm upon CsA binding with minimal broadening (78 Hz). TfmW-CypA^121^ electroporated cells showed similar resonances for free and bound states (Figure 3E). Intriguingly, in HEK293T cells expressing tfmW-CypA^121^ by transient transfection, we observed a considerably larger line width of the tfm resonance (860 Hz, Figure 3F), centered around +0.3 ppm downfield of the equivalent resonance in electroporated cells. This observation implies that the intracellular tfmW-CypA^121^, when transiently expressed, has ample time to engage in specific or nonspecific interaction with other cellular partners. Also, interactions may be different for tfmW-CypA^121^ due to the larger tfm group in the tfmW residue. Gratifyingly, the ^19^F resonance of CsA-complexed tfmW-CypA^121^ sharpens (560 Hz) and resides at the same frequency as that observed for the complex in buffer and after electroporation.

In summary, our current work establishes a mammalian cell-directed GCE system for the site-specific incorporation of tfmF and tfmW with efficiency between 80% and 100% of wild-type protein expression using the *Ec*TyrRS/tRNA_CUA_ and *Ma*PylRS/tRNA_CUA_ pairs, respectively. To our knowledge, this is the first demonstration of tfm-ncAA encoding in mammalian cells. We demonstrate that both fluorinated amino acids are efficiently incorporated into proteins, using the highly abundant cytoplasmic protein CypA. Although transfection-based in-cell NMR may pose its own specific challenges, the mammalian cell-directed GCE system expands the in-cell NMR toolkit by providing a viable alternative for particularly challenging proteins, where recombinant protein expression from bacterial systems for electroporation is limited. Our general platform broadens access to in-cell ^19^F NMR for real-time studies of protein dynamics, ligand binding, and intracellular protein interactions.

## Supplementary Material



## Notes

Material and Methods, GCE tool development and validation, in-cell NMR methodology, NMR data acquisition and processing, chemical synthesis of trifluoromethyl tryptophan, ^1^H, ^13^C, and ^19^F NMR spectra, and LC-MS data (PDF)
