# Supplementary material for: Endogenous Site-Specific Encoding of Trifluoromethyl-Bearing Phenylalanine and Tryptophan for in-Cell 19F NMR
Source: J Am Chem Soc. 2026 Feb 13;148(7):6744–9. doi: 10.1021/jacs.5c18349 (PMC12951447; doi:10.1021/jacs.5c18349)
Supplement: Supplementary file 1 [file ja5c18349_si_001.pdf]

Supporting Information

Endogenous site-specific encoding of trifluoromethyl-bearing phenylalanine and tryptophan for in-cell ^{19}F NMR

George Augustin,[1+] Fatema Bhinderwala,[2+] Nathan D Alexander,[1] Iker Hernández,[3] Stanislau Stanisheuski,[1] Christina M. Monnie,[2] Alex J Eddins,[1] Yogesh M. Gangarde,[1] Vadim A. Soloshonok, [3,4] Mikel Oiarbide, [3] Aitor Landa,[3] Richard B Cooley,[1] Angela M Gronenborn,[2*] Ryan A Mehl [1*]

[1] Department of Biochemistry and Biophysics, 2011 Agricultural and Life Sciences, Oregon State University, Corvallis, Oregon 97331, United States

[2] Department of Structural Biology, University of Pittsburgh School of Medicine, 3501 Fifth Ave., Pittsburgh, PA 15261, United States

[3] Department of Organic Chemistry I, Faculty of Chemistry, University of the Basque Country UPV/EHU, Paseo Manuel Lardizabal 3, 20018 Donostia-San Sebastián, Spain

[4]IKERBASQUE, Basque Foundation for Science, Bilbao 48011, Spain

+ Authors contributed equally to the work

***Corresponding authors AMG and RAM;** Email: amg100@pitt.edu and ryan.mehl@oregonstate.edu

Table of contents:

Supplementary Methods

1. Synthesis and characterization of trifluoromethyl tryptophan (tfmW)
2. Selection and optimization of ncAA-specific amino-acyl tRNA synthetases (aaRSs)
3. In-cell NMR Spectroscopy

List of Figures:

Figure S1: Chemical Synthesis of tfmW

Figure S2: Selection of tfmW-specific synthetase

Figure S3: Efficiency, fidelity, and expression of tfmW-sfGFP^{150} across *E. coli* expression hosts

Figure S4: Genetic encoding of tfmW into sfGFP^{150} in HEK293T cells

Figure S5: Genetic encoding of tfmF into sfGFP^{150} in HEK293T cells

Figure S6: Site-specific introduction of tfm ncAAs into CypA^{60} and CypA^{W121} in HEK293T cells

Figure S7: Top-down mass spectrometry for mammalian CypA^{WT}

Figure S8: Mass spectrum for tfmF-CypA^{F60}

Figure S9: Mass spectrum for tfmW-CypA^{W121}

Figure S10: Full spectral window of the in-cell spectra of tfmF-CypA^{F60} and tfmW-CypA^{W121}

List of Tables:

Table 1: List of primers used for cloning sfGFP^{WT}, sfGFP^{150}, CypA^{WT}, and CypA$^{\#}$ in pUC, pIRE4, pet41, and pAcBac1 vectors

Table 2. Description of the plasmids used for tfmF and tfmW incorporation in HEK293T cells

Table 3: Sequence of CypA^{WT} and CypA^{TAG} plasmid inserts used for tfmF and tfmW incorporation.

Table 4: Plasmids used for tfmF and tfmW incorporation in *E.coli*

Table 5: Summary of purified protein yields for tfmF and tfmW labeling from *E. coli*

Supplementary Methods

1. **Chemical Synthesis for tfmW**

General methodology for the synthesis and characterization of trifluoromethyl tryptophan.

All non-aqueous reactions were performed in an inert atmosphere using oven-dried glassware and were stirred magnetically. Yields refer to chromatographically purified and spectroscopically pure compounds, unless otherwise stated. Heat-requiring reactions were carried out using a hot plate with an oil bath and a condenser. Reactions requiring low temperatures were performed using cooling bath circulators (Huber T100E) and isopropanol baths. Organic layers were washed with aqueous phases and then dried over $MgSO_4$ and filtered through filter paper. Organic solvents were evaporated under reduced pressure using Büchi R-210 rotary evaporators.

Solvents and reagents

Reagents were purchased from various commercial suppliers (e.g., Merck, Across, BLDpharm), stored as specified by the manufacturers, and used without prior purification. Et_3N was purified by distillation over KOH. CH_2Cl_2 and CH_3CN were dried over calcium hydride (CaH_2).

Chromatography

Reactions and fractions from flash chromatographic columns were monitored by thin-layer chromatography (TLC) on Merck silica gel 60 F254 plates and visualized by fluorescence quenching under UV light. In addition, TLC plates were stained with a dipping solution of potassium permanganate (1 g) in 100 mL of water (limited lifetime), followed by heating. Chromatographic purification was performed using ROCC 60 silica gel (40-63 µm) as the stationary phase and a suitable mixture of solvents (see Experimental Procedures for more details) as the eluent.

Melting points

Melting points were determined in open capillaries in a Stuart SHP3 melting point apparatus.

NMR spectra

^{1}H NMR and ^{13}C NMR spectra were recorded using 300 MHz, 400 MHz, 500 MHz or 600 MHz and 126 MHz or 151 MHz spectrometers, respectively. Chemical shifts (δ) are quoted in parts per million referenced to DMSO-d_6 (δ = 2.50), CD_3OD (δ = 3.31), and D_2O (δ = 4.79) for ^{1}H NMR and relative to the central resonances of CD_3OD (δ = 49.0) and DMSO-d_6 (δ = 39.5) for ^{13}C NMR. ^{19}F spectra of compounds **4**, **5,** and **6** were recorded at 298K on a Bruker Avance Neo 400 spectrometer, equipped with Iprobe, at 376 MHz using fluorobenzene as internal reference (-113.05 ppm). These spectra were processed using MestReNova software. The multiplicity of each signal is described using the following abbreviations: s, singlet; d, doublet; dd, doublet of doublets; dt, doublet of triplets; ddd, doublet of doublets of doublets; q, quartet; m, multiplet. Coupling constants (J) are reported in Hertz (Hz).

Mass spectra

MS spectra were recorded on an ESI-ion trap Mass spectrometer (Agilent 1100 series LC/MSD, SL model). Mass spectrometry analysis was performed in the General Research Service (SGIker) of the University of the Basque Country (UPV/EHU).

IR spectra

Infrared spectra were measured employing a Bruker ALPHA-P compact FT-IR spectrometer.

Synthesis of starting materials:

Compound **3** was purchased from BLDpharm and used without further purification. Compounds **1**[1] and **2**[2] are known compounds and were synthesized following reported experimental procedures.

2.1. Ethyl propiolate-promoted addition of 2-benzylthioimidazolone 2 to gramine 1

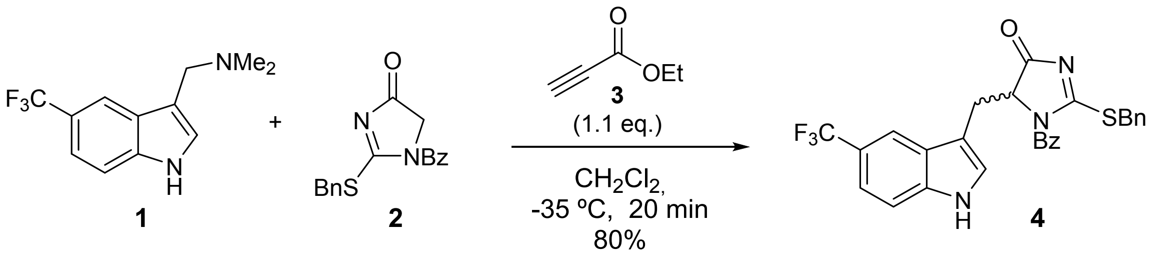

To a solution of gramine **1** (1.50 g, 6.19 mmol) in dry CH_2Cl_2 (60 mL) at room temperature, 2-benzylthioimidazolone **2** (3.84 g, 12.38 mmol, 2 eq.) was added. After the solution is cooled to -40 °C, ethyl propiolate **3** (0.67 g, 6.81 mmol, 1.1 eq.) was added, and the reaction mixture was stirred at the same temperature for 10 minutes. Afterwards, the mixture was allowed to reach to a temperature of -35 °C slowly over 30 minutes. Then, the solvent was evaporated under reduced pressure, and the crude compound was purified directly by column chromatography (eluent: hexane/ethyl acetate from 5:1 to 1:1) to obtain **4** as a white foam. Yield 2.51 g, 4.95 mmol, 80%. ^{1}H NMR (500 MHz, DMSO-d_6) δ 11.41 (d, J = 2.5 Hz, 1H), 7.84 – 7.17 (m, 13H), 7.08 (d, J = 2.3 Hz, 1H), 5.17 (dd, J = 5.9, 3.3 Hz, 1H), 4.30 (d, J = 13.5 Hz, 1H), 4.23 (d, J = 13.5 Hz, 1H), 3.20 (dd, J = 15.2, 3.3 Hz, 1H), 2.64 (dd, J = 15.2, 5.8 Hz, 1H). ^{13}C NMR (126 MHz, DMSO-d_6) δ 184.8, 184.8, 166.9, 137.4, 136.0, 133.3, 132.5, 129.0, 128.9, 128.5, 127.7, 127.5, 126.4, 126.1, 125.6 (q, J = 271.3 Hz), 119.5 (q, J = 31.0 Hz), 117.5 (q, J = 3.7 Hz), 116.1 (q, J = 4.3 Hz), 112.2, 107.3, 64.8, 36.4, 25.4.^{19}F NMR (376 MHz, DMSO-d_6) δ –58.37 (s, 1H). HRMS (ESI) m/z: $[M+Na]^+$ Calcd. for $C_{27}H_{20}F_3N_3O_2SNa$* 530.1121; Found 530.1124. IR (cm^{-1}): 1727, 1676, 1431, 1324, 1252, 1154, 1104, 900, 806, 699, 665.

Hydrolysis of 4 into Fluorinated Hydantoin 5

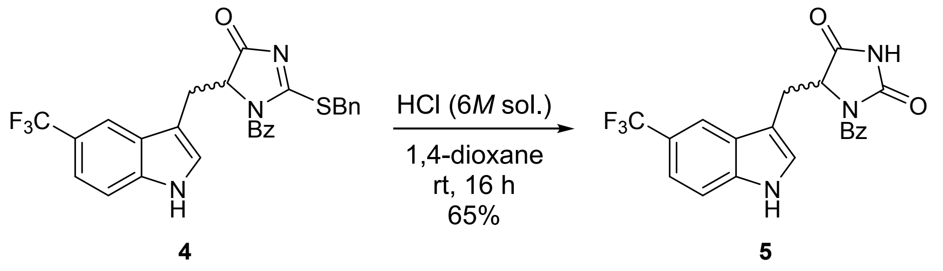

A solution of aq. HCl 6 M (9.1 mL, 54.5 mmol, 11 eq.) was added dropwise to a solution of imidazolone **4** (2.51 g, 4.95 mmol) in 1,4-dioxane (75 mL) at 0 °C. Once the addition was complete, the reaction was stirred at room temperature overnight. Afterward, the mixture was cooled to 0 °C, and a saturated solution of $NaHCO_3$ was added until a basic pH was reached. The aqueous layer was extracted with CH_2Cl_2 threefold, the combined organic layers were dried over $MgSO_4$, and the solvent was evaporated under reduced pressure. The crude product was purified by silica gel flash column chromatography (from CH_2Cl_2 to CH_2Cl_2/MeOH 99:1) followed by recrystallization in CH_2Cl_2 to obtain hydantoin **5** as a white solid, m.p.: 226–230 °C. Yield: 1.29 g, 3.22 mmol, 65%. ^{1}H NMR (300 MHz, Methanol-d_4) δ 7.74 (dt, J = 1.8, 0.9 Hz, 1H), 7.56 – 7.09 (m, 9H), 5.14 (dd, J = 5.8, 2.3 Hz, 1H), 3.75 (dd, J = 15.0, 5.8 Hz, 1H), 3.60 – 3.50 (m, 1H). ^{13}C NMR (126 MHz, MeOD) δ 174.7, 170.3, 154.9, 139.2, 135.5, 132.8, 129.9, 128.5, 128.4, 127.4, 126.8 (q, J = 270.2 Hz), 122.5 (q, J = 31.3 Hz), 119.2 (q, J = 3.2 Hz), 117.5 (q, J = 4.5 Hz), 113.0, 109.7, 62.7, 24.6. ^{19}F NMR (376 MHz, DMSO-d_6) δ –58.64 (s, 1H). HRMS (ESI) m/z: $[M+Na]^+$ Calcd. for $C_{20}H_{14}F_3N_3O_3Na$* 424.0879; Found 424.0884. IR (cm^{-1}): 3305, 1735, 1647, 1367, 1324, 1101, 807, 670, 599, 464.

Conversion of Hydantoin 5 into trifluorinated Tryptophan 6

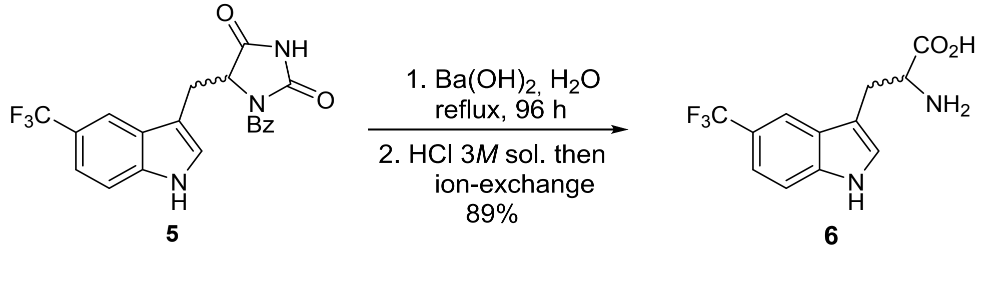

To a solution of hydantoin **5** (1.29 g, 3.22 mmol, 1 eq.) in 35 mL H_2O at 0 °C (ice bath), $Ba(OH)_2$ monohydrate (2.44 g, 12.88 mmol, 4 eq.) was added. The resulting mixture was refluxed for 96 hours, during which the evolution of ammonia gas and the formation of a barium carbonate precipitate were observed. The mixture was then cooled down to room temperature, and the precipitate was filtered and washed with water. The aqueous layer was acidified with 3 M HCl (6.4 mL, 19.3 mmol, 6 equiv.). Then, water and the volatiles (HCl) were removed under reduced pressure in the rotary evaporator, followed by final removal under a vacuum pump. The resulting

solid was dissolved in water and then loaded into a dowex® 50WX2 hydrogen form (50–100 mesh) ion exchange resin (previously washed with 1.0 M HCl). Afterwards, the resin was treated with water until the pH of the eluent was neutral. Then, the compound was liberated from the resin by eluting with a 5% aqueous solution of NH_3. The obtained basic solution was concentrated under vacuum to yield 5-CF_3-Trp **6** in its zwitterionic form as a white solid, m.p. = 237–241 °C. Yield: 0.78 g, 2.87 mmol, 89%. ^{1}H NMR (400 MHz, D_2O) δ 8.12 (dt, *J* = 1.9, 0.9 Hz, 1H), 7.65 – 7.61 (m, 1H), 7.53 (dd, *J* = 8.6, 1.7 Hz, 1H), 7.42 (s, 1H), 4.05 (dd, *J* = 8.0, 4.8 Hz, 1H), 3.50 (ddd, *J* = 15.4, 4.9, 0.9 Hz, 1H), 3.32 (dd, *J* = 15.4, 8.1 Hz, 1H). ^{13}C NMR (151 MHz, MeOD) δ 174.5, 139.7, 127.9, 127.5, 127.1 (q, J = 270.4 Hz), 122.4 (q, J = 31.5 Hz), 119.2 (q, J = 3.5 Hz), 117.3 (q, J = 4.4 Hz), 112.9, 110.9, 56.6, 28.1. ^{19}F NMR (376 MHz, DMSO-d_6) δ –58.15 (s, 1H). HRMS (ESI) m/z: $[M+H]^+$ Calcd. for $C_{12}H_{12}F_3N_2O_2$ 273.0845; Found 273.0844. IR (cm^{-1}): 3254, 1572, 1397, 1334, 1301, 1159, 1121, 894, 809, 666, 552, 422.

^{1}H and ^{13}C and ^{19}F NMR spectra

^{1}H NMR (500 MHz, DMSO-d_6) of **4**:

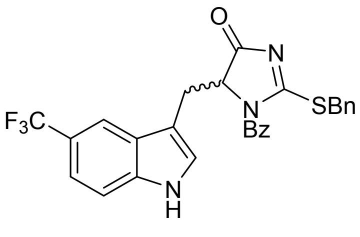

4

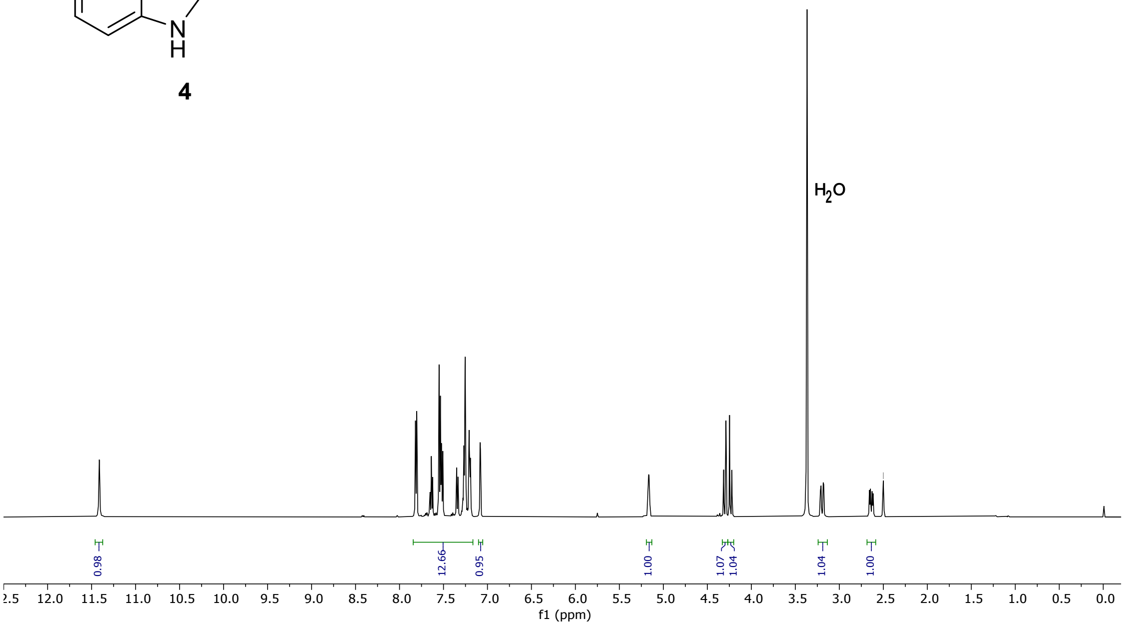

$^{13}C\{^1H\}$ NMR spectrum (126 MHz, DMSO-d_6) of **4**

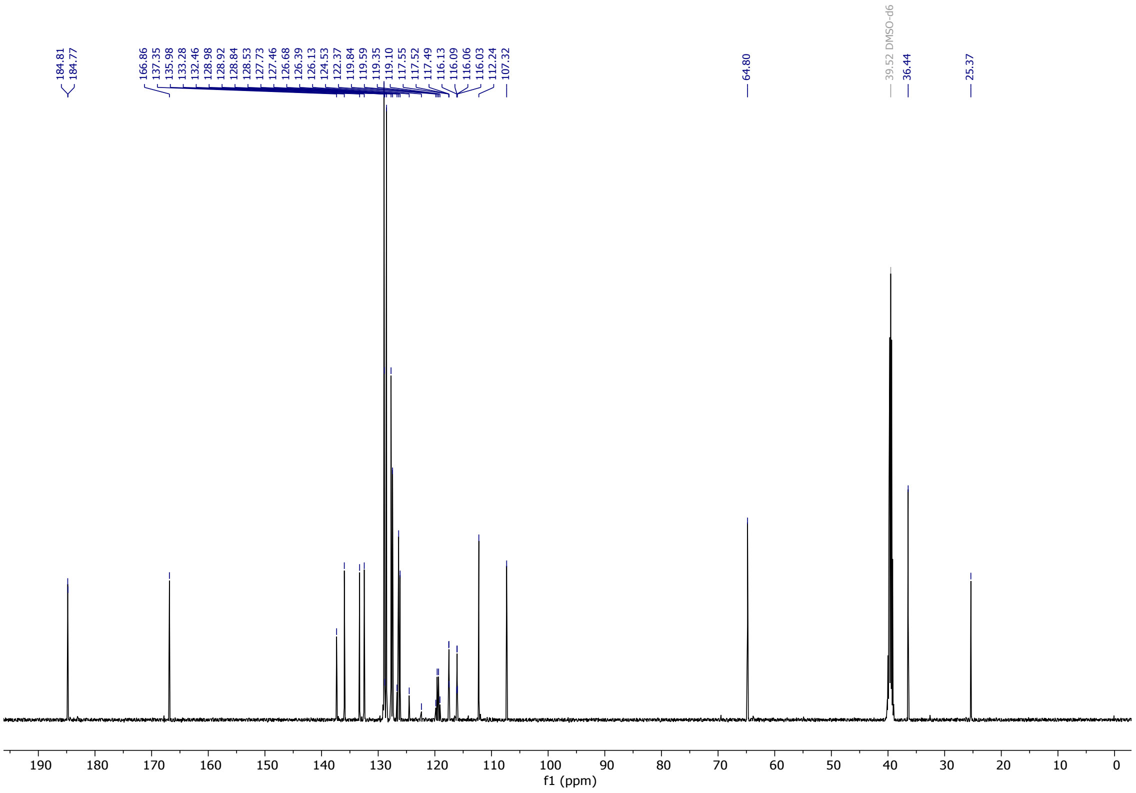

^{19}F NMR spectrum (376 MHz, DMSO-d_6) of **4**

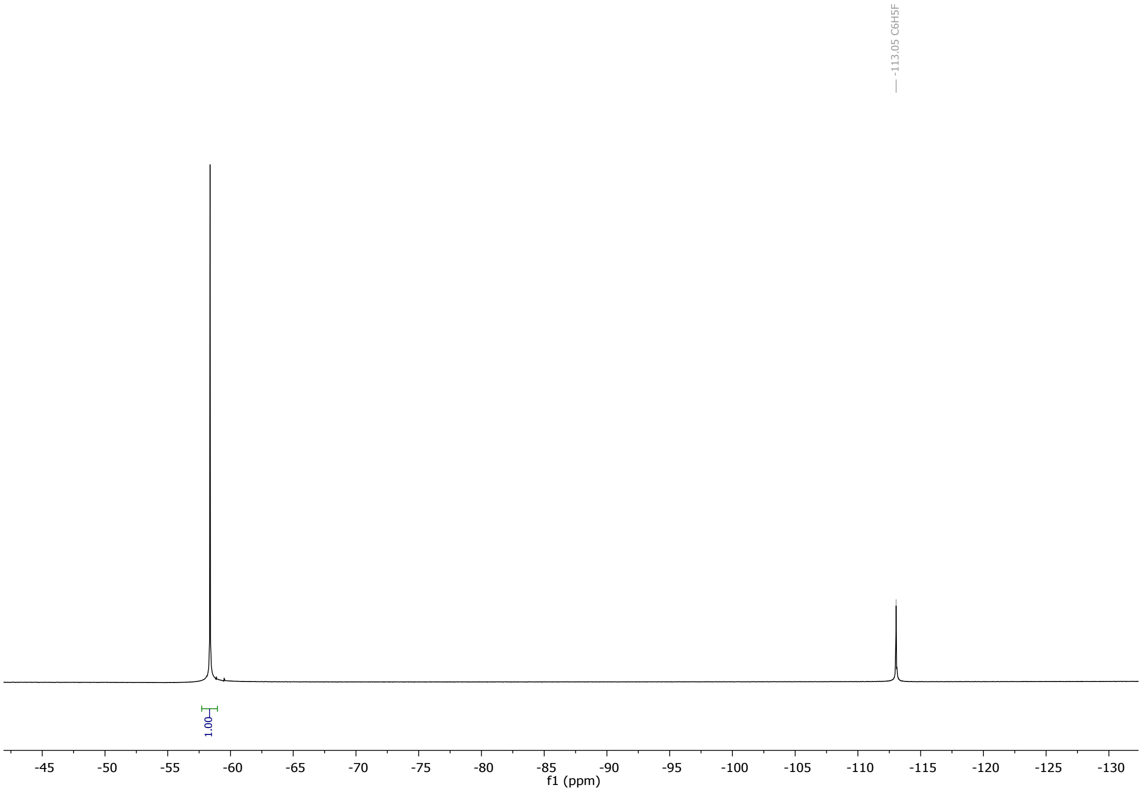

^{1}H NMR spectrum (300 MHz, CD_3OD) of **5**:

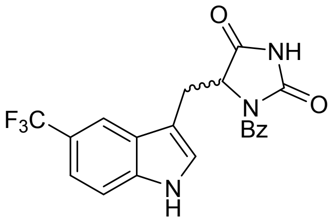

5

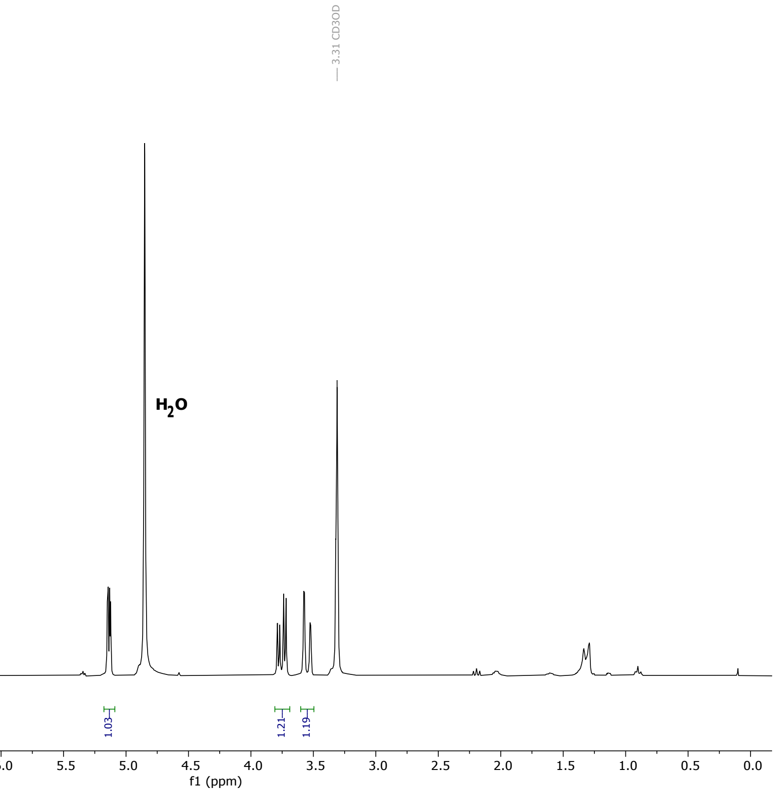

$^{13}C\{^{1}H\}$ NMR spectrum (126 MHz, CD_3OD) of **5**

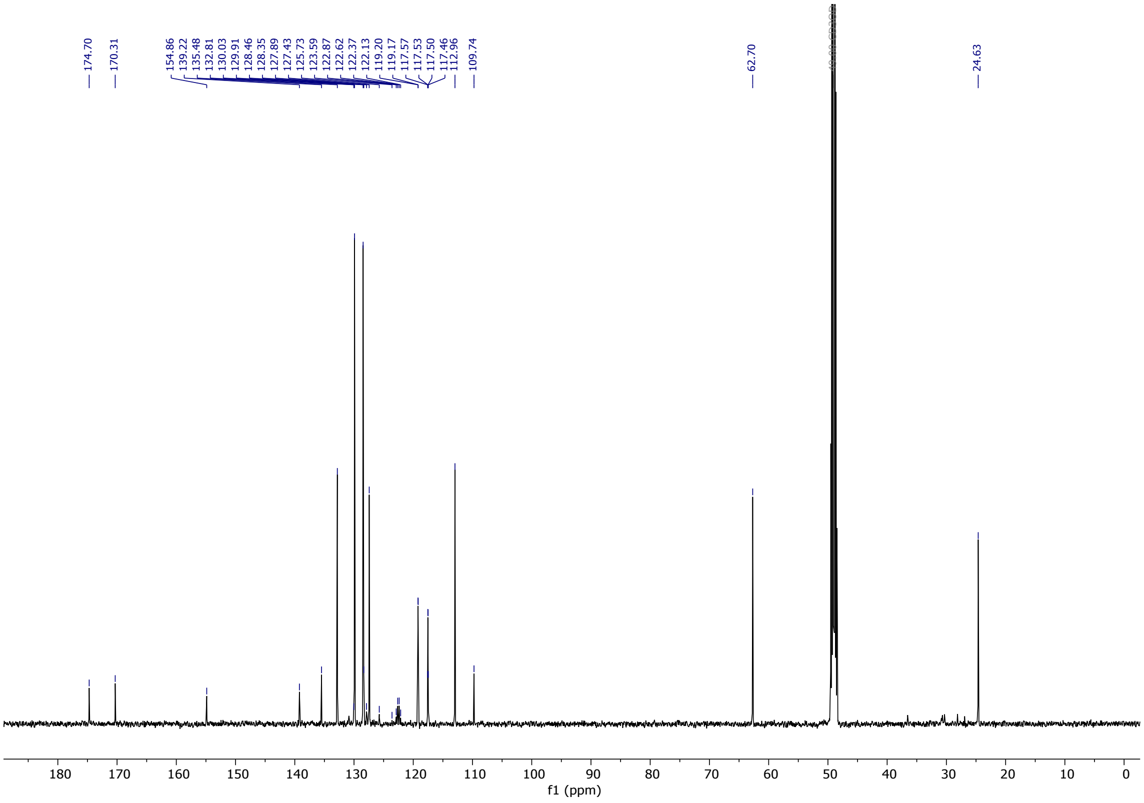

^{19}F NMR spectrum (376 MHz, DMSO-d_6) of **5**

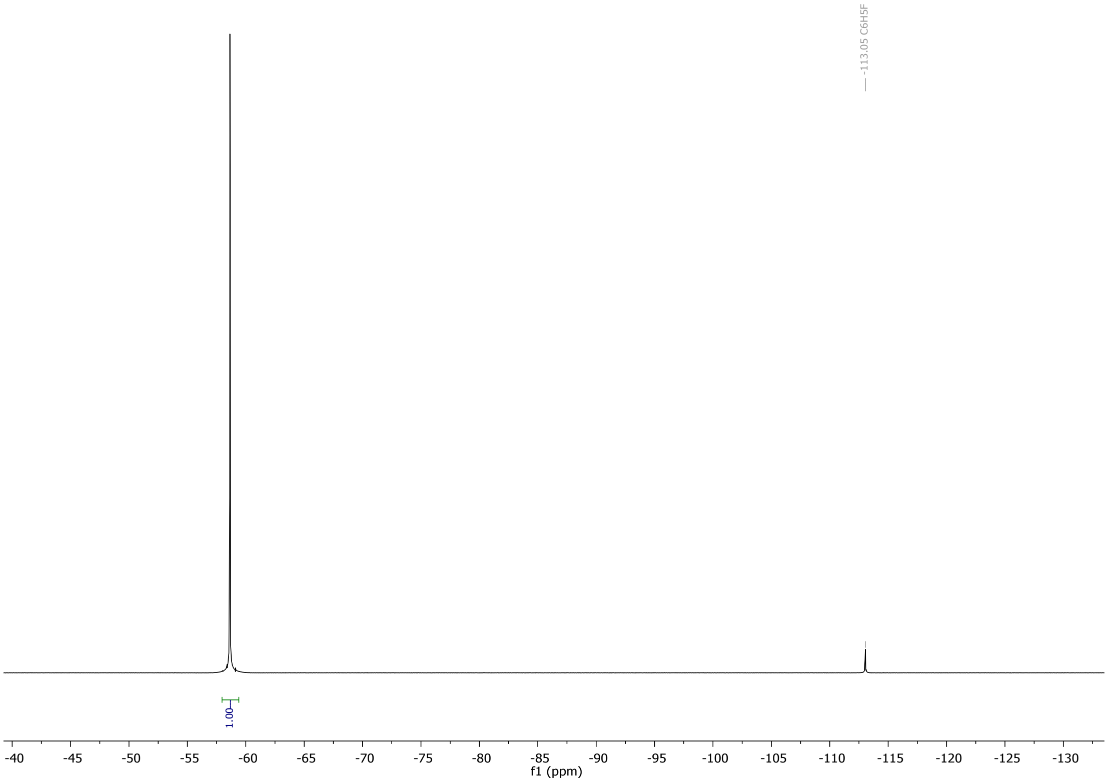

^{1}H NMR spectrum (400 MHz, D_2O) of **6**:

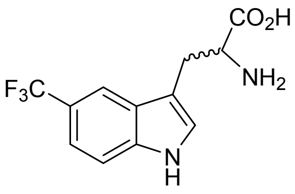

6

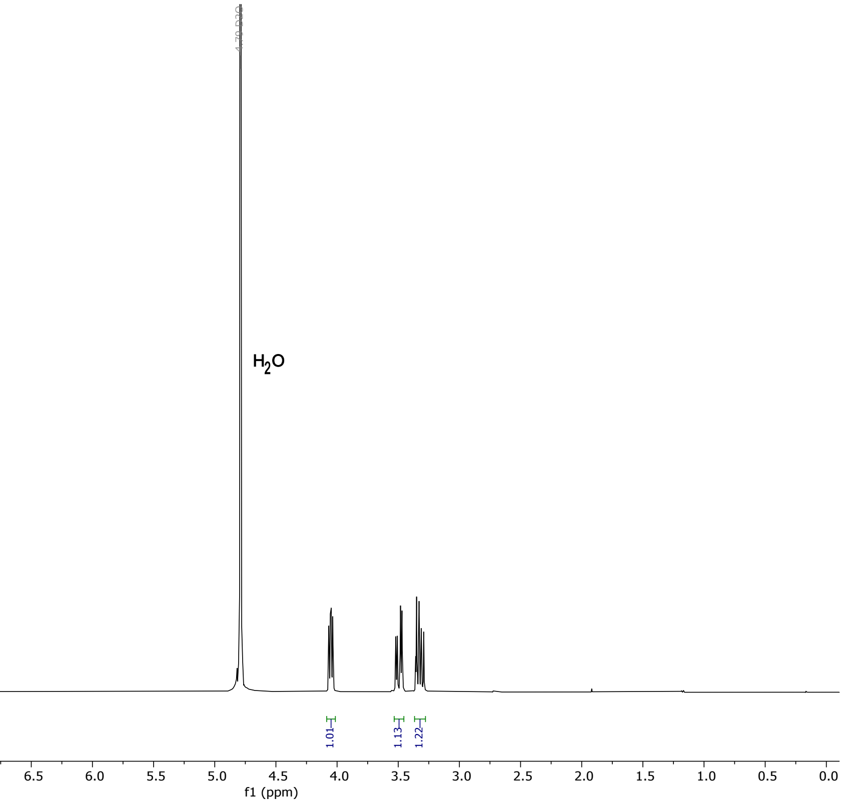

$^{13}C\{^{1}H\}$ NMR spectrum (151 MHz, CD_3OD) of **6**:

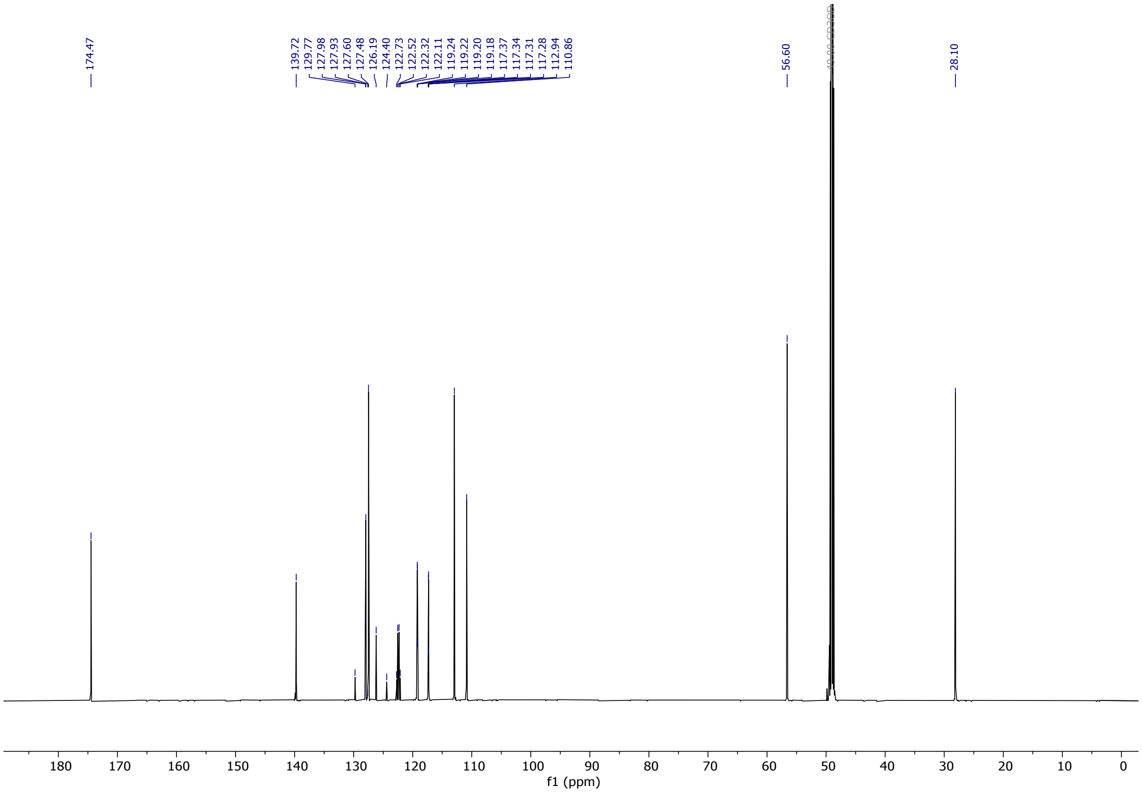

^{19}F NMR spectrum (376 MHz, DMSO-d_6) of **6**:

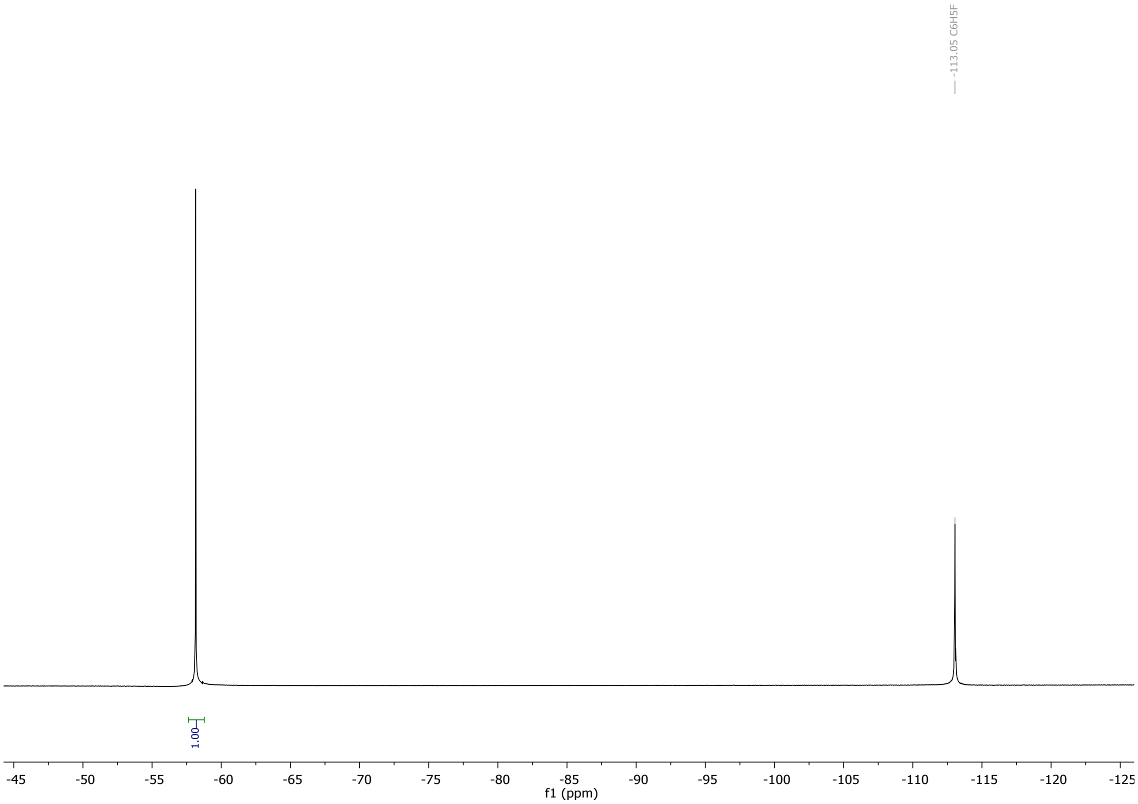

^{1}H-^{13}C HSQC NMR (600 MHz - 151 MHz, CD$_3$OD) of **6**:

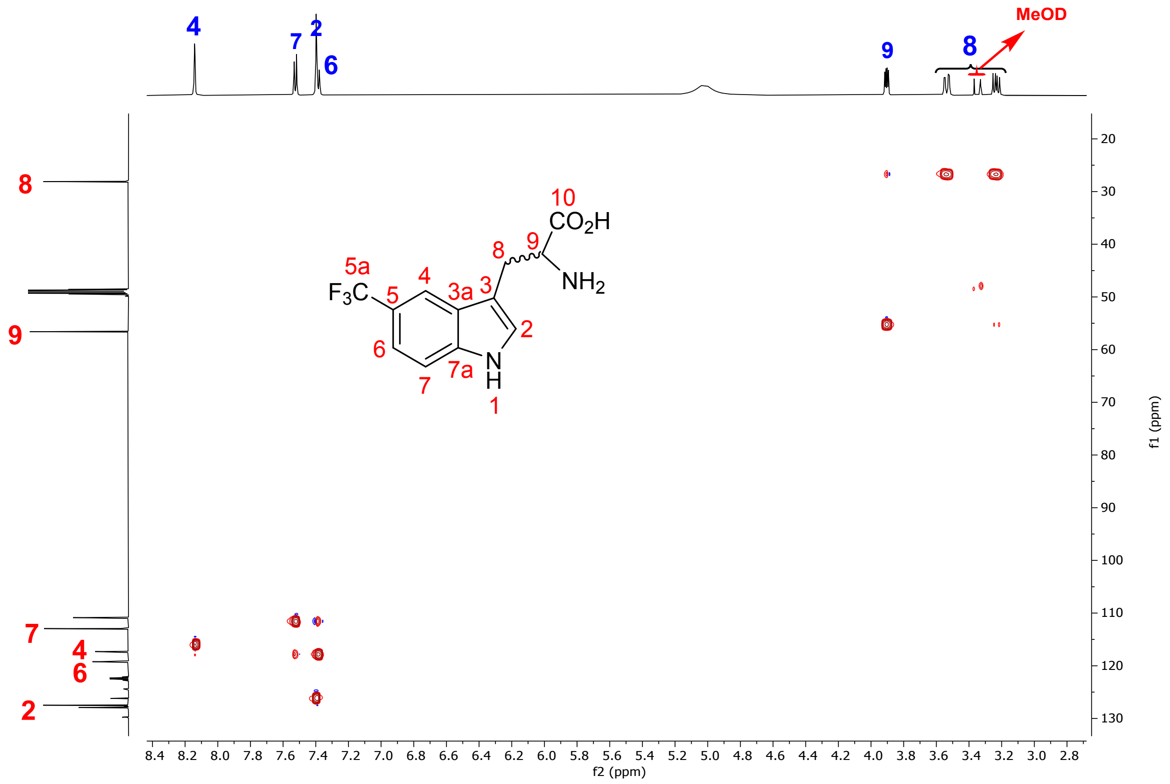

2D ^{1}H-^{13}C HMBC NMR spectrum (600 MHz - 151 MHz, CD_3OD) of **6**

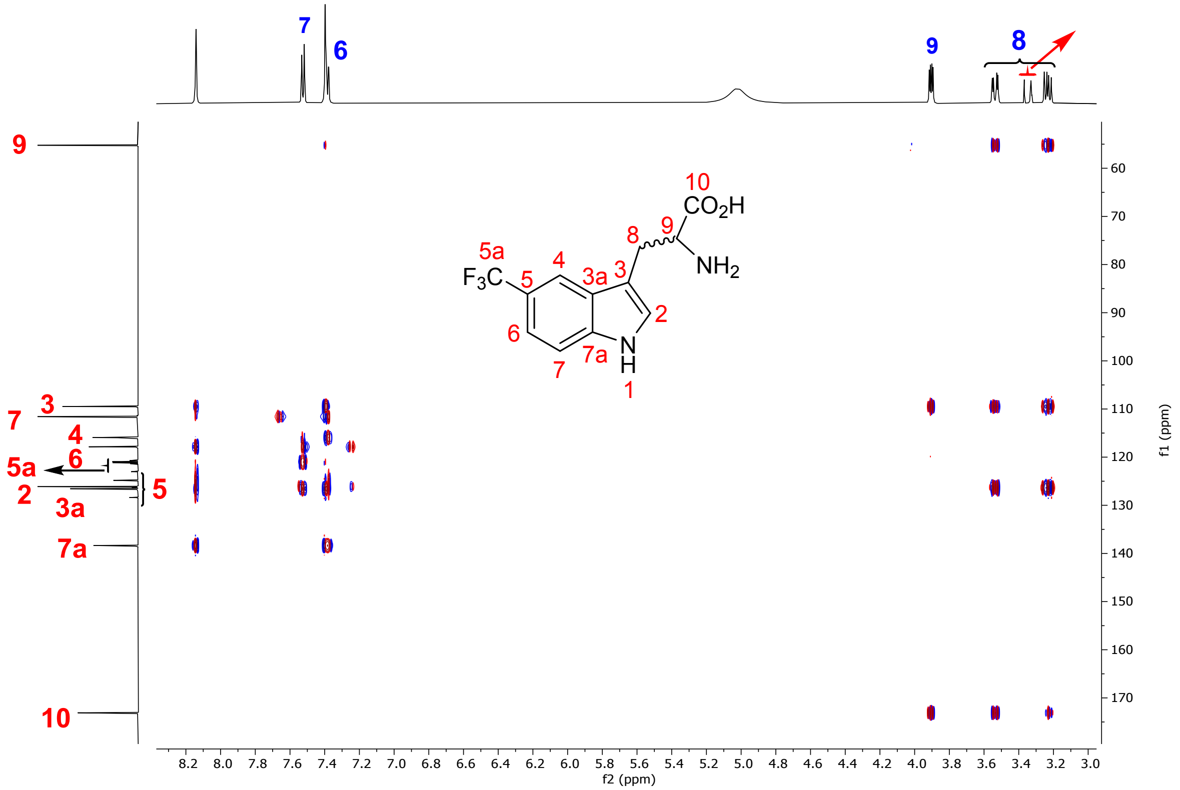

4. High Resolution Mass Spectra (HRMS)

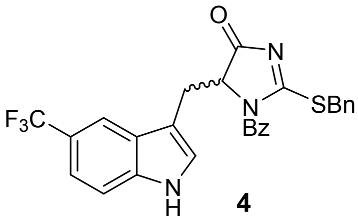

4

HRMS (ESI) m/z: $[M+Na]^+$ Calcd. for $C_{27}H_{20}F_3N_3O_2SNa$* 530.1121; Found 530.1124.

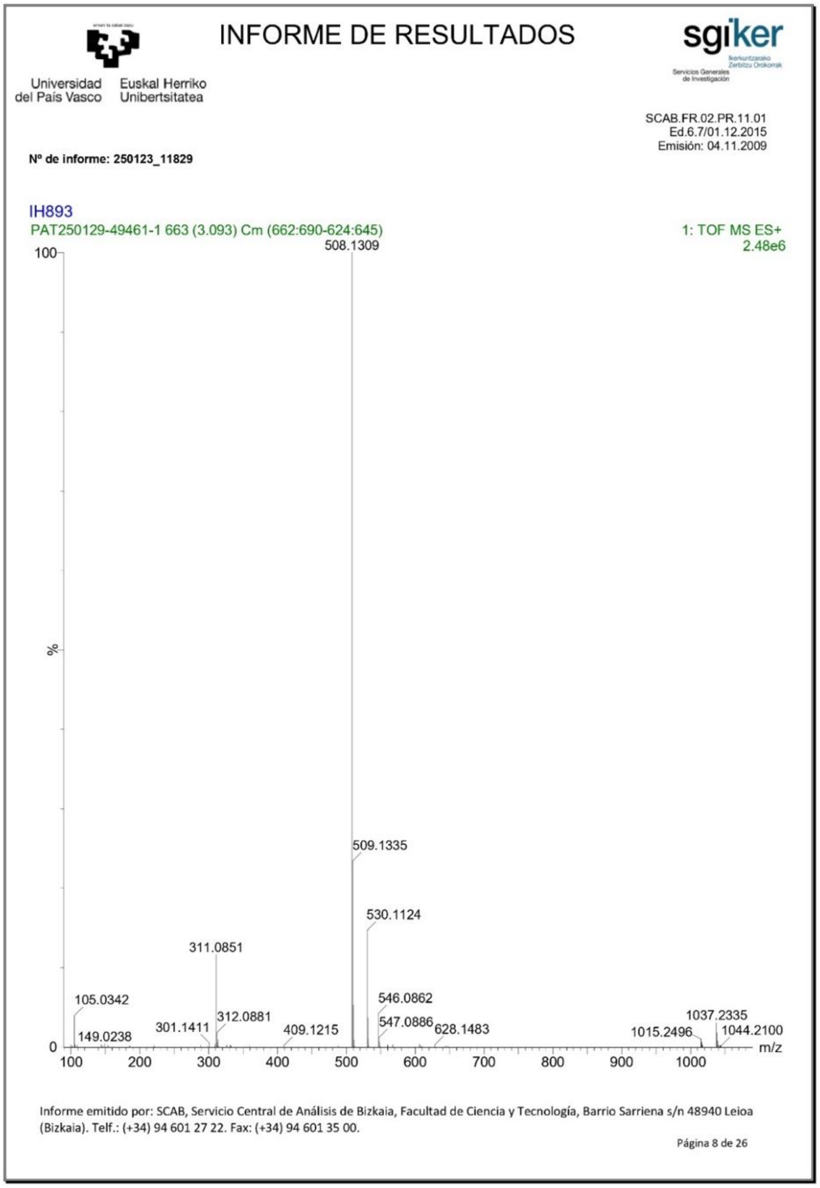

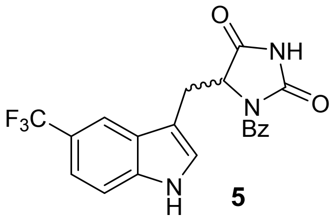

5

HRMS (ESI) m/z: $[M+Na]^+$ Calcd. for $C_{20}H_{14}F_3N_3O_3Na$* 424.0879; Found 424.0884.

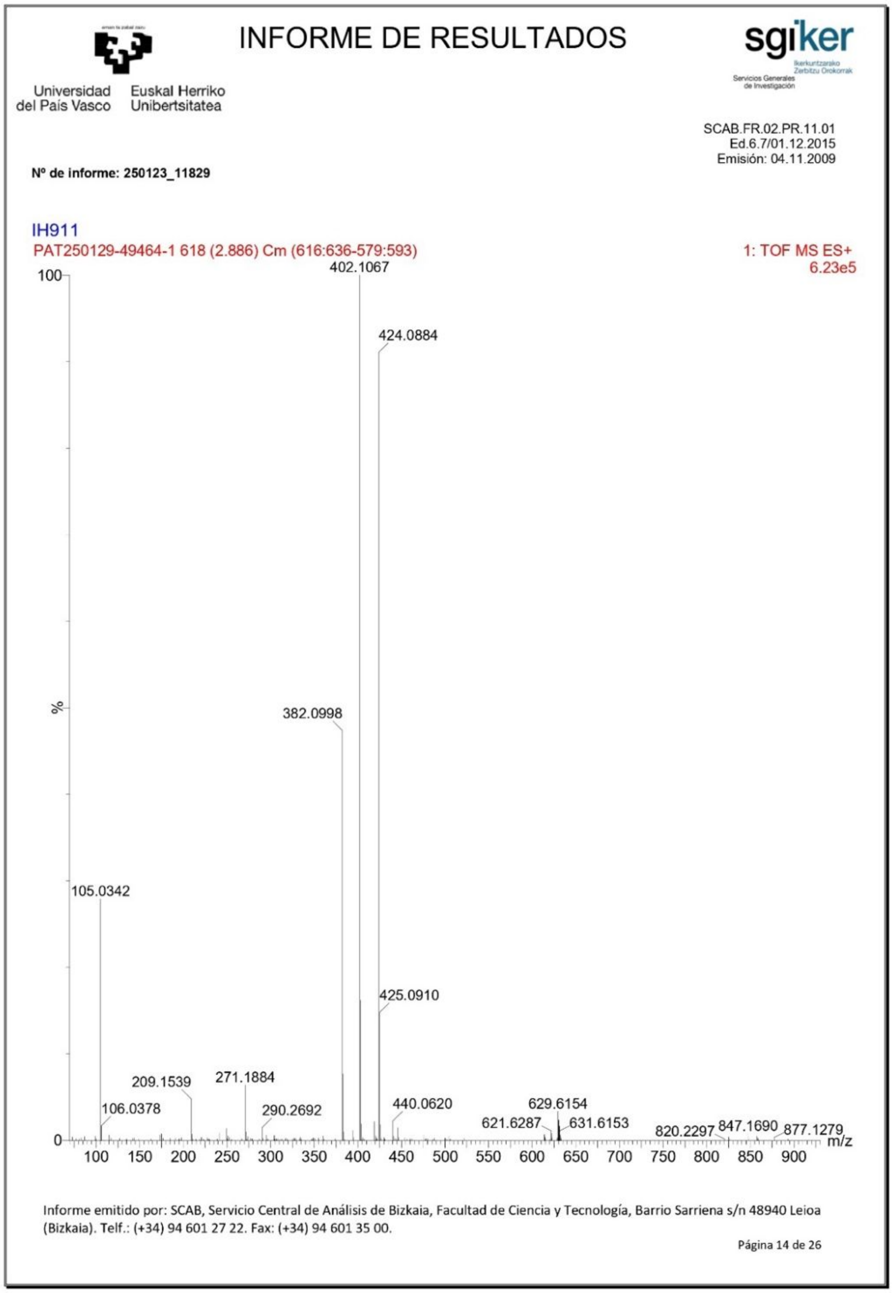

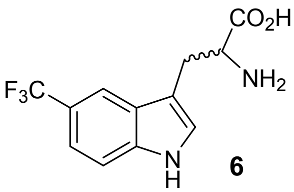

6

HRMS (ESI) m/z: $[M+H]^+$ Calcd. for $C_{12}H_{12}F_3N_2O_2$ 273.0845; Found 273.0844.

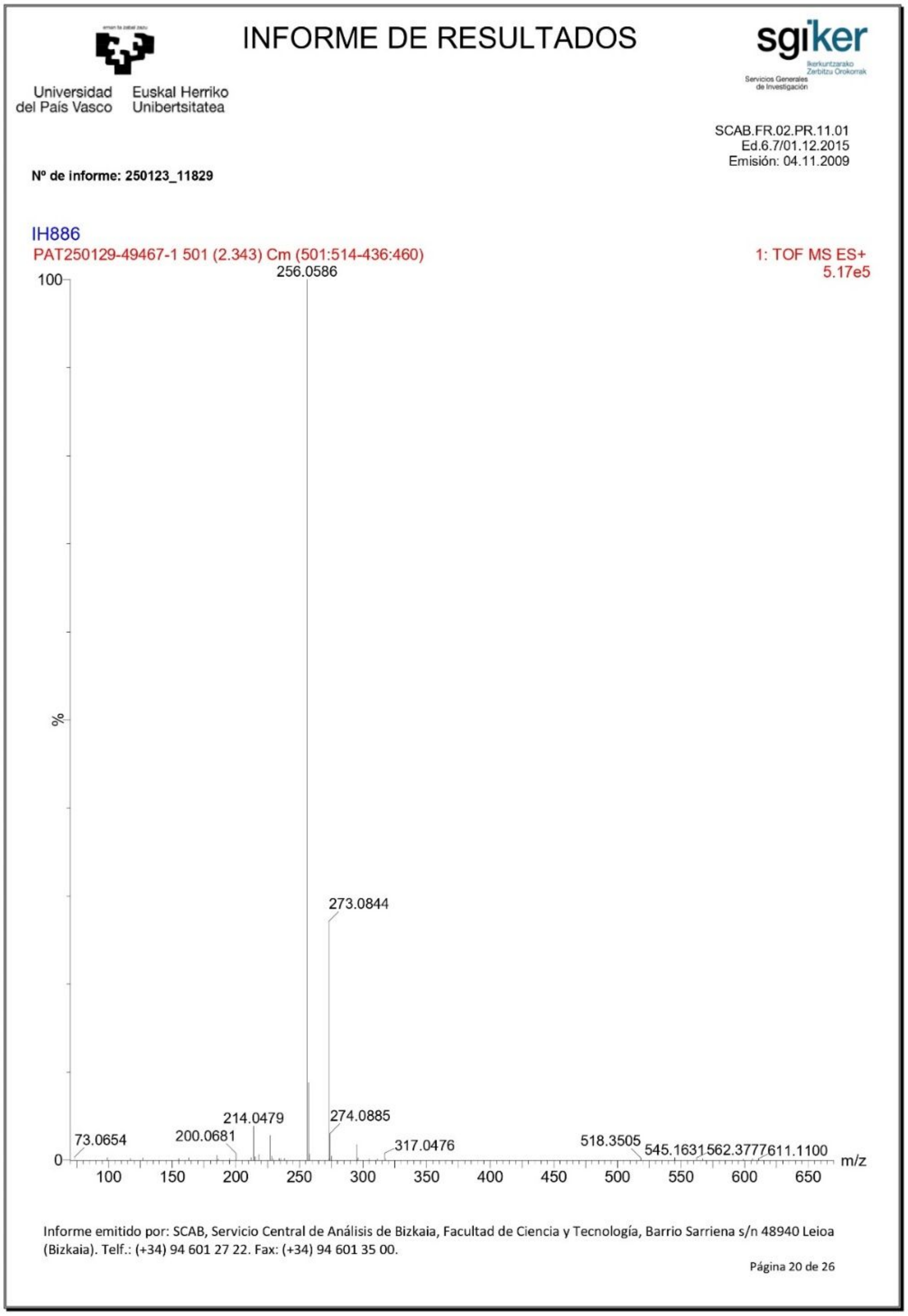

2. Selection and optimization of ncAA-specific amino-acyl tRNA synthetases (aaRSs) and mammalian cell expression of ncAA-labeled proteins

Selection of ncAA-specific amino-acyl tRNA synthetases (aaRSs)

For all noncanonical amino acids (ncAAs) aaRS selections, we carried out a standard double-sieved life-death selection[1] using a previously created 3.2 x 10^6 member pBK plasmid library

encoding a complete set of mutations of 5-active site residues in the *Methanomethylophilus alvus* (*Ma*) pyrrolysine tRNA synthetase (PylRS) (Figure S2A-C).[3]

Library propagation and positive selection round: A *Ma* pBK plasmid library (containing the aaRS gene) was propagated, and a positive selection round was performed using Ma pREP (a positive selection plasmid with a TAG site under the chloramphenicol acetyltransferase gene) with 400 μM tfmW and 1 mM tfmF. A lower concentration of tfmW was used since only limited amounts of tfmW were available. A pBK plasmid library obtained from the positive selection (pBK-pPos) step for the tfm-ncAA was used for the negative selection round.

Negative selection round: A negative selection round was performed using purified *Ma* pBK-Pos1 library plasmid paired with *Ma* pNeg (a negative selection plasmid) using (0.2%) L-arabinose as a selection pressure. The pBK plasmid library obtained from the negative selection (pBK-Neg1) step for the respective tfm-ncAA was used for the fluorescence-based selection.

Fluorescence-based cell sorting: The remaining library was transformed into DH10B cells containing a TAG-interrupted fluorescent reporter gene. Electrocompetent DH10B (50 μL) were co-transformed with 5-15 ng of purified *Ma* pBK-Neg1 library plasmid obtained from the life/death selection round and 15 ng of pALS3 plasmid (encoding sfGFP^{150TAG}). For the tfmW selection, 400 μM tfmW was provided for the fluorescence-based cell sorting in autoinduction media (AIM). Using fluorescence-activated cell sorting (FACS), the top-most fluorescent 5% cells, cultured using autoinduction media[3] with 400 μM tfmW, were collected (Figure S2D, E) and further cultured under the same conditions. Of these, the top-most fluorescent 45% cells were collected. These were grown on fluorescence-inducing plates with 400 μM tfmW. The 96 most fluorescent colonies were each grown in liquid culture with and without 400 μM tfmW (Figure S3A). Of these, nine individual RSs were sequenced and shown to encode the same RS phenotype (Figure S3B, C). Expression of tfmW-sfGFP in *E. coli* was confirmed by whole protein mass spectroscopy (Figure S3D-F). This RS was cloned into the pAJE1-90-*Ma* PylRS nitroY/haloY-F5 plasmid (Addgene: 225684) by substituting the nitroY/haloY-F5 RS with the tfmWRS-A2 evolved in this study. tfmW was dissolved in a 400 mM stock solution using deionized (DI) water and an equimolar amount of NaOH before use in cultures. For the tfmF selection, DH10B cells, transformed with *Ma* pBK Neg1 and pALS3, were grown on fluorescence-inducing plates with 1 mM tfmF, and the 96 most fluorescent colonies were grown in liquid culture supplemented with 1 mM tfmF. No RSs selective for tfmF were identified.

Molecular cloning for tfmW-protein expression in mammalian cells

The tfmWRS, the reporter genes, sfGFP and sfGFP^{150TAG}, and the CypA^{WT} and CypA^{TAG}genes were cloned into the pAcBac1 vector backbone containing *Ma* tRNAs.[4] sfGFP^{WT}-His$_6$ (reporter gene with a C-terminal His$_6$ tag) and sfGFP^{150TAG} (reporter gene having a C-terminal His$_6$ tag used for tfmW encoding), CypA ^{WT}-His$_6$ (with a C-terminal His$_6$ tag), CypA^{WT}-FLAG (with a C-terminal FLAG tag), CypA^{W121TAG}-His$_6$ (with a C-terminal His$_6$ tag) for encoding tfmW,

$CypA^{W121TAG}$-FLAG (with a C-terminal FLAG tag), and *Ma*RS tfmWA2 (*Ma* tfmW RS gene) were cloned into the pAcBac1 vector containing 4×*Ma*-$tRNA^{Pyl}$ using a combination of touchdown PCR and restriction digestion with the Fast Digestion (FD) restriction enzymes *NheI* and *EcoRI* (Thermo Fisher scientific, USA). Ligation was performed overnight at 16 °C in a thermocycler using T4 DNA ligase (New England Biolabs Inc.). *E. coli* NEB Stable cells were transformed with the ligation mixture and plated onto LB agar supplemented with ampicillin (100 µg/mL). Individual colonies were picked, and 5 mL of XYT medium containing ampicillin (100 µg/mL) was inoculated for plasmid production. The plasmid sequences were confirmed by restriction digestion and sequencing (services provided by Plasmidsaurus, USA).

Mammalian optimization of tfmW-protein expression

Before transfection, HEK-293T cells were seeded at ~40% confluency in 24-well plates containing fresh, prewarmed Corning DMEM (Dulbecco's Modified Eagle's Medium) and grown to ~70–80% confluency. Transfections were performed using 600 ng total DNA per well, following the manufacturer's instructions for JetPrime reagent (PolyPlus, VWR, USA). To determine the optimal concentration of tfmW for incorporation into mammalian cells, tfmW was supplied at concentrations ranging from 50 µM to 1 mM. A 100 mM stock solution of tfmW was prepared in water by adding eight equimolar equivalents of NaOH, followed by dilution into 1 M HEPES buffer to adjust the pH to 7.4 for use during transfection. For these experiments, the plasmid pair pAcBac1-$sfGFP^{150TAG}$ 4×*Ma*-$tRNA_{CUA}$ and pAcBac1-*Ma*RS-tfmW 4×*Ma*-$tRNA_{CUA}$ was co-transfected in an 8:1 ratio, as reported previously for the pAcBac1 vector[5]. Prior to adding the DNA complexes, the culture medium was replaced with fresh DMEM supplemented with 10% FBS to minimize the inhibition by secreted factors. DNA mixtures were prepared in 0.5 mL DMEM containing the indicated concentration of tfmW and added to the cells. Following transfection, cells were incubated for 48 hours at 37 °C in a 5% CO_2 atmosphere to allow for reporter expression. Mean fluorescence intensity (MFI) was measured using flow cytometry.

To determine the optimal plasmid ratio for tfmW encoding, the two plasmids, pAcBac1-sfGFP-^{150TAG}-4×*Ma*-$tRNA_{CUA}$ and pAcBac1-*Ma*RS tfmW 4×*Ma*-$tRNA_{CUA,}$ were co-transfected at ratios ranging from 1:1 to 20:1 in the presence of 100 µM tfmW, by increasing the amount of pAcBac1-$sfGFP^{150TAG}$ while keeping pAcBac1-*Ma*RS tfmW 4×*Ma*-$tRNA_{CUA}$ constant. Following transfection, cells were incubated for 48 h at 37 °C in a 5% CO_2 atmosphere to allow reporter expression, and mean fluorescence intensity (MFI) was quantified by flow cytometry.

Molecular cloning for tfmF-protein expression in mammalian cells

The polyspecific *Ec*TyrRS from pAcBac3-OMeYRS-8xYtR-EGFP-3TAA-152TAG-TEV-3xUAA was cloned into the pIRE4 plasmid[6] , replacing its specific *Ec*TyrRS, generating the pGA2-GK-Ec-OMeYRS-4xtRNA. Molecular cloning of EF1α-$sfGFP^{WT}$-His_6 and EF1α-$sfGFP^{150TAG}$-His_6 into the pGA2-GK-Ec-OMeYRS-4xtRNA plasmid, generating pGA1-EF1α-sfGFP150TAG-4xtRNA, which contains four copies of *E. coli* Tyr (*Ec*Tyr) $tRNA_{CUA,}$ was performed using a combination of touchdown PCR and restriction–ligation cloning methods. Prior to cloning, EF1α-sfGFP was amplified from a pUC57 plasmid containing an EF1α-sfGFP. The PCR-amplified product and pIRE4 vector were digested with *BamHI* and *HindIII*, and ligation was

carried out overnight at 16 °C using T4 DNA ligase (New England Biolabs Inc.) in a thermocycler. *E. coli* NEB Stable cells were transformed with the ligation mixture and plated on LB agar containing kanamycin (50 µg/mL). 5 mL of XYT medium supplemented with kanamycin (50 µg/mL) was inoculated with individual colonies and grown for plasmid isolation. Positive clones were confirmed by restriction digestion and sequencing (services provided by Plasmidsaurus, USA).

As described above, $CypA^{WT}$ and its $CypA^{TAG}$ variants (F60TAG, F88TAG, F113TAG, W121TAG, and F145TAG) were constructed. Briefly, gene fragments of $CypA^{WT}$ and its variants were synthesized as gBlocks (Integrated DNA Technologies, Coralville, USA) and cloned into pUC57-EF1α cassettes (named pUC-P2) that contain the EF1α promoter using the SLiCE method.[7] *E. coli* DH10B cells were transformed with the resulting constructs, and colonies were grown in LB medium at 37 °C. For cloning into the pIRE4 vector, the pIRE4-*E. coli* TyrRS 4×$tRNA_{CUA}$ plasmid was digested with *MluI* and *EcoRI* (FD restriction enzymes, Thermo Fisher Scientific). Plasmids encoding $\textit{cypA}^{WT}$ and its variants were amplified from the pUC-P2 plasmid by touchdown PCR, digested with *MluI* and *EcoRI*, and ligated into the digested pIRE4-GCE plasmid. Ligation mixtures were incubated overnight at 16 °C before being transformed into *E. coli* NEB Stable cells. 5 mL of XYT medium supplemented with kanamycin (50 µg/mL) was inoculated with individual colonies and grown for plasmid isolation. Positive clones were confirmed by restriction digestion and sequencing. (services provided by Plasmidsaurus, USA)

To generate FLAG-tagged constructs, primers incorporating a FLAG sequence at the C-terminus were designed. DNA sequences encoding $CypA^{WT}$ and its variants were amplified by touchdown PCR, digested with *EcoRI* and *HindIII*, and ligated into the pIRE4 plasmid digested with the same enzymes. Ligation reactions were incubated overnight at 16 °C before being transformed into *E. coli* NEB Stable cells.

5 mL of XYT medium supplemented with kanamycin (50 µg/mL) was inoculated with individual colonies and grown for plasmid isolation. Positive clones were confirmed by restriction digestion and sequencing. (services provided by Plasmidsaurus, USA)

Optimization of tfmF encoding with the *Ec*TyrRS/$tRNA_{CUA}$ pair.

To assess tfmF and pAzF encoding levels, HEK293T cells were transfected using the GFP reporter plasmids and the pIRE4-*Ec*TyrRS plasmid following the protocol for tfmW encoding above. For tfmF incorporation, pUC-P2 ($sfGFP^{WT}$ and $sfGFP^{150TAG}$) and pGA2-GK-Ec-OMeYRS-4xtRNAwere co-transfected at a 1:1 molar ratio (600 ng total DNA per well). ncAAs (tfmF and pAzF) were supplied, ranging from 50 µM to 1 mM, from stock solutions prepared in 1M HEPES-buffered water (pH 7.4). Transfected cells were incubated for up to 24-48 h at 37 °C and supplemented with 5% CO_2, and GFP expression was monitored every 24 h by fluorescence microscopy (Keyence BZ-X800, Keyence Corporation, USA).

Plasmid ratio optimization was performed using the same workflow described above for determining the optimized amino acid concentration. In this case, pGA1-EF1α-sfGFP-4xtRNA ($sfGFP^{WT}$ and $sfGFP^{150TAG}$) and pGA2-GK-Ec-OMeYRS-4xtRNAwere co-transfected at ratios

ranging from 1:1 to 20:1 in the presence of 500 μM tfmW, by varying the amount of the $sfGFP^{150TAG}$ construct while keeping the *Ma*RS tfmW plasmid constant. Cells were incubated for 48 hours at 37 °C in a 5% CO_2 atmosphere, and reporter expression was assessed by flow cytometry to determine the mean fluorescence intensity (MFI). The resulting plasmids have been deposited at the Addgene plasmid repository; plasmid nos. 251517, 251518, 251519, and 251520.

Fluorescence Microscopy and Flow Cytometry Assessment

Prior to flow cytometry, $tfmGFP^{150TAG}$ (tfmF and tfmW) and pAzF-GFP^{150TAG} expression in transfected cells was first confirmed by fluorescence microscopy using a Keyence BZ-X800 fluorescence microscope (Keyence Corporation, USA). Flow cytometry was performed on a CytoFLEX instrument (Beckman Coulter), and data were analysed with CytExpert software version 2.2. Before analysis, cells were washed and resuspended in Dulbecco's phosphate-buffered saline (DPBS), lacking Ca^{2+} and Mg^{2+}, and 10,000 events were recorded per sample. Data were plotted using GraphPad Prism software. Non-transfected cells and cells cultured without the ncAA served as controls; a minimal basal level of GFP expression was detected in these samples. For every expression and flow cytometry assessment, control plasmids ($sfGFP^{WT}$) were included to ensure accurate comparison and validation of reporter expression.

Western blot analysis of tfmW-CypA and tfmF-CypA expression

pAcBac1-GOI-FLAG (GOI-$CypA^{W121TAG}$) and pIRE4-GOI-FLAG (GOI-$CypA^{TAG}$ constructs) plasmids were co-transfected with their respective synthetase/tRNA plasmids - pAcBac1-*Ma*RStfmW for tfmW incorporation and pIRE4-*Ec*TyrRS for tfmF incorporation, enabling site-specific ncAA incorporation into the proteins described above, using the methods in 12-well plates containing HEK293T cells. Cells were allowed to grow for 48 hours at 37 °C and supplemented with 5% CO_2. After 48 hours of transfection, the cells were washed with 1x Dulbecco's Modified Phosphate buffer saline (DPBS) lacking Ca^{2+} and Mg^{2+} ions and then detached using trypsin-EDTA. Cells were then centrifuged and lysed by adding RIPA buffer (Sigma, USA) containing protease inhibitor (Roche tablets). Before running SDS-PAGE, a BCA assay was used to determine the protein concentration according to the manufacturer's instructions, utilizing BSA protein standards for calibration. Fractions of cell lysates were resolved by 12% SDS-PAGE at 120 V using a Bio-Rad SDS-PAGE apparatus. Proteins from SDS-PAGE were transferred to an Immobilon PVDF membrane using the Western transfer apparatus (Bio-Rad) at 25 V for 30 min. PVDF membrane was blocked with 1:1 Intercept (TBS) protein-free blocking buffer in TBS-T buffer (20 mM Tris-HCl, 150 mM NaCl, pH 7.6, and 0.1% Tween-20) overnight. After incubation, the PVDF membrane was thoroughly rinsed with TBS-T buffer. Then, 1:1000 dilutions of monoclonal anti-FLAG M2 antibody (Millipore Sigma) were added to the membrane, which was incubated for 2 hours. After washing the membrane with TBS-T buffer, IR Dye 800 CW goat anti-mouse IgG secondary antibody (Thermo Fisher Scientific, USA) was added to the membrane and incubated for 1 hour. The membrane was washed three times with TBST buffer before detecting the protein signal using a ChemiDoc MP imaging system (Bio-Rad, USA). Expression levels of $CypA^{WT}$ and $CypA^{w121TAG}$ were normalized to those of GAPDH.

Expression and purification of tfmF- and tfmW-proteins from HEK293T cells

To purify the $sfGFP^{WT}$-His_6, $tfmsfGFP^{150TAG}$-His_6, $CypA^{WT}$-His_6, $CypA^{W121TAG}$-His_6, and $tfmCypA^{F60TAG}$-His_6 proteins from HEK293T cells, transfection was scaled up to 100 mm plates according to the recommended protocol for the Jet Prime reagent (PolyPlus) (VWR, USA). The total concentration of DNA used for each reaction was 10-12 µg of DNA. A predetermined ratio of pAcBac1 plasmids (containing the RSs: the expression gene of interest CypA or sfGFP) was used, as indicated in the transfection section. Cells were incubated for 48 hours after adding transfection reagents and ncAA-amino acids. After 48 hours of expression, cells were harvested by centrifugation at 400 g for 5 minutes at 4 °C. Pelleted cells were resuspended in a minimal (5 mL) volume of DPBS containing Ca^{2+} and Mg^{2+} ions, and supplemented with protease inhibitors (PMSF, NaVO4, and PPi cocktails) for 30-45 minutes on ice. The cells were then lysed using a probe sonicator (10 seconds on, 10 seconds off, with 20% amplitude) and centrifuged at 12,000 g for 10 minutes. The cell supernatant was collected, diluted with wash buffer (50 mM Tris, 500 mM NaCl, 5 mM imidazole, pH 8.0) in a 1:2 ratio, and then loaded onto Ni-NTA resin for 1.5 hours to bind at 4 °C. The bound resin was washed with 5 to 6 column volumes of wash buffer to remove unbound protein before elution in 50 mM Tris, 500 mM NaCl, 250 mM imidazole, pH 8.0. After elution, proteins were further desalted by gel filtration on a gravity 10/300 Superdex S75 column (Cytiva Life Sciences) in 50 mM ammonium bicarbonate buffer, pH 6.4. Purified proteins were confirmed by SDS-PAGE analysis.

Mass spectrometry analysis of tfmW- and tfmF-proteins

Proteins were concentrated to 2 mg/mL using a 10 kDa cut-off filter (Millipore, USA), and the concentration was determined using Nanodrop (Thermo Fisher Scientific, USA). Proteins purified from HEK293T cells were diluted to ~600 µg/mL in 50 mM ammonium bicarbonate buffer. The pH was adjusted to 6.4 for CypA and pH 7.4 for sfGFP, prior to mass spectrometry analysis. Samples were centrifuged at 12,000 × g for 10 minutes and transferred to LC vials. Agilent Infinity II Bio LC was used for on-column buffer exchange. 20 µL of ~40 µg/mL samples were injected on a NativePac OBE-1 SEC column (2.1 mm x 50 mm, 3 µm, 80 Å) and eluted with 0.1% formic acid at 60 µl/min. Intact protein mass data were acquired on a 6545XT Advance Bio Q-TOF system. The VCap parameter was set to 5500 V, the nozzle Voltage to 2000 V, the fragment voltage to 300 V, and the skimmer voltage to 140 V. Sheath and drying gas flow rates were set to 11 L/min with temperatures set to 350 °C and 400 °C, respectively. Deconvolution was performed in Agilent MassHunter BioConfirm 12.0. The Maximum Entropy deconvolution algorithm was used in the range 10-50kDa with a mass step of 1.0 Da. The baseline subtraction factor was set to 7.00. The average mass was calculated for the top 25% of peak intensity. MS graphs were plotted using GraphPad Prism software (GraphPad Prism Inc., USA).

The tfmF and tfmW labeling efficiency for CypA variants prepared for in-cell NMR was also assessed by mass spectrometry. ESI LC-MS measurements were performed at 1µM protein concentration on a Bruker Q-TOF instrument, using a reverse-phase AdvanceBio peptide guard column (Agilent Technology), where mobile phases A and B comprised 5% acetonitrile with 0.01% FA and 80% acetonitrile and 0.01% FA, respectively. The resulting LC-MS spectra were processed using Bruker Compass Software, and the MS data were processed using Maximum Entropy-based deconvolution to obtain the M+ ion mass for each sample. The instrument was calibrated using the ESI Low Tuning mix I (Agilent Technology) to a 1.0 ppm mass % difference before each use.

Top-down mass spectrometry

All parameters for the 6545XT Advance Bío Q-TOF system were set as described above. The ExD cell tuning was performed using carbonic anhydrase to optimize the efficiency of MS2/ECD modes. Based on the spectra acquired during the intact mass analysis, the top 5 charge states were selected for fragmentation in Targeted MS/MS mode. The resulting spectra were processed using ExD Viewer 4.6.28 with default parameters.[8] Variable acetylation was set for all lysines and the N-terminus of the CypA^{WT} protein, and variable modification search was used. The results of the variable modification search were manually inspected and confirmed.

3. In cell NMR spectroscopy

Expression and purification of CypA from *E. coli* for in-cell NMR

The cDNAs encoding the T2Q/F60TAG and T2Q/W121TAG CypA mutants were ordered as gBlocks™ for Gibson assembly (Integrated DNA Technologies, Coralville, USA). The gBlocks™ were inserted into the pET41 vector using NdeI and XhoI sites, and the DNA sequence was verified by sequencing. BL21(DE3) cells were transformed with plasmids encoding tRNA and cognate tRNA synthetase for tfmF and tfmW labeling. The pDule2 plasmid for tfmF [9] or the tfmWRSA2 in the pAJE plasmid for tfmW, and the CypA encoding plasmid for protein expression. The details regarding plasmids and inserts are summarized in Tables 3 and 4. Essentially, protein expression and purification methods were as described in previous work.[10]

For tfmF and tfmW incorporation into CypA, 100 mg/L of tfmF (Sigma, USA) and tfmW (custom synthesized) were added to the cell culture at an optical density A_{600} of 0.8. Cells were then cooled for 30 minutes, following which the expression was induced by 0.5 mM isopropyl-β-d-thiogalactopyranoside. After 18 hours of growth at 16°C, cells were harvested by centrifugation at 4000 g for 20 minutes at 4°C. The resulting cell pellet was resuspended in 35 mL/L SP A buffer (25 mM sodium phosphate, 2 mM DTT, pH 5.8), and cells were lysed by sonication (5 s on and 5 s off at 50% power level). The cell lysate was centrifuged at 18,000 g for 30 min. The pH and conductivity of the supernatant were adjusted to ensure they were below pH 6 and 2.5 mS/cm, respectively, and loaded onto a cation exchange column (HiTrap SP column (Cytiva, Chicago, IL), followed by elution with a 0-1 M NaCl gradient in SP B buffer (25 mM sodium phosphate, 1M NaCl, 2 mM DTT, pH 5.8). CypA-containing fractions eluted over a range of 12-18% SP B buffer. These fractions were pooled and concentrated using a 10 kDa Amicon filter, followed by size exclusion chromatography on a Superdex 75 column (Cytiva, Chicago, IL), which was equilibrated in NMR buffer (20 mM sodium phosphate, 150 mM NaCl, 1 mM TCEP, 0.2% NaN_3, pH 7.0). Proteins for electroporation into HEK293T cells were dialyzed into electroporation buffer (100 mM sodium phosphate, 15 mM magnesium chloride, 15 mM HEPES, 5 mM potassium chloride at pH 7.0)

In-cell NMR sample preparation by electroporation of exogenous protein in HEK293T cells

tfmF60 CypA and tfmW121 CypA variants were delivered by electroporation into HEK293T cells in two independent experiments. The in-cell CypA (Sample 1, Figure 3 B and C) and matched CsA binding study (Sample 2, Figure 3 E and F) were performed as two consecutive NMR experiments. For each electroporation, 120 million cells were harvested and resuspended in 1.0 mL of electroporation buffer (containing 2 mM reduced glutathione and 2 mM ATP) with 2.0 mM

tfmF60/W121 CypA. Using the NEPAgene® electroporator system, cells were split into 12 cuvettes, each containing 110 µL (10 million cells each) of cell suspension, and electroporated twice with a ~ 30 s interval. The electroporation program was previously tested and optimized for the HEK293T cells to obtain 50% cell survival post-electroporation with high concentrations of protein. Immediately after electroporation, 1 mL of prewarmed DMEM (Gibco, Life Technologies) was added to each cuvette, and cells were gently suspended before being transferred into prewarmed DMEM in T175 flasks for cell recovery over four hours. Post recovery, cells were washed three times with prewarmed Dulbecco's Phosphate Buffered Saline (DPBS; Gibco Life Technologies) to remove detached dead cells. A total of 56 million cells (Sample 1) were harvested, resuspended in 1.0 mL L-15 medium containing 10% FBS (Gibco, Life Technologies) and 10% D_2O, and transferred to 5 mm NMR tubes. After the initial in-cell NMR data were acquired, the cells were spun down gently at 50 g for 5 minutes to prepare the supernatant sample, which was transferred into a fresh 5mm NMR tube. This sample is necessary to ensure that no protein leakage occurred during the NMR experiment. The supernatant controls show no protein leakage, or free amino acid after NMR data acquisition. This is an essential control for robust and reliable ^{19}F in-cell NMR measurements and should always be carried out.

For the CsA binding study, the cell pellet was resuspended in 40 mL of L-15 medium, containing 10% FBS and 50 µM CsA, and incubated for 30 minutes at 37 °C. The cells were gently spun down at 50 g for 5 minutes and resuspended in 1mL of L-15 medium, containing 10% FBS, 50 µM CsA, and 10% D2O. They were then transferred to a 5 mm NMR tube for the NMR experiment. Cyclosporine A was purchased from Sigma-Aldrich (St. Louis, MO, USA) and dissolved in d6-DMSO and prepared as a 50 mM stock solution. To ensure that CsA was fully dissolved in the L-15 medium, the L-15 medium was stirred for 2 hours at 4 °C before use.

Before each in-cell NMR experiment, the cells in the NMR tube were gently centrifuged at 30 g for 5 minutes to ensure that the cells were packed and located within the NMR detection coil, minimizing any changes in ^{19}F signal intensity due to cell settling in the NMR tube. Additionally, a dummy experiment was collected for the first 30 minutes to allow enough time for the cells to settle into the NMR detection coil before final data acquisition.

In-cell NMR sample preparation by transient transfection using GCE in HEK293T cells

tfmF-CypA^{60} and tfmW-CypA^{121} variants were expressed by transient transfection of HEK293T cells with the respective plasmids in two independent experiments.

For in-cell NMR, transfection was scaled up to 4 T75 flasks per experiment, according to the recommended protocol for the Jet Prime reagent (PolyPlus) (VWR, USA). The total concentration of DNA used for each reaction was 12 µg of DNA. A predetermined ratio of pAcBac1 plasmids (RS: expression gene of interest) was used (1:1 and 1:8), as indicated in the transfection section. Cells were incubated for 48 hours after adding transfection reagents and 500 µM tfm amino acids. After 48 hours of expression, cells were washed three times with DPBS to ensure that the in-cell NMR samples contained no free tfm amino acids. The cells were harvested by trypsinization followed by centrifugation at 50 g for 10 minutes at 4°C. Cells were counted using Nucleocounter (Chemometyx, USA), and 56 million cells were used for each in-cell experiment. The in-cell CypA and CypA-CsA binding experiments were performed as described above.

^{19}F NMR spectroscopy for in-cell NMR in HEK293T cells

All ^{19}F spectra were recorded on a 14.1 T Bruker AVANCE spectrometer, equipped with a CP TXO F/C-H-D triple-resonance, z-axis gradient cryoprobe. The Larmor frequencies of ^{1}H and ^{19}F are 600.1 and 564.6 MHz, respectively. ^{19}F chemical shifts were referenced to trifluoracetic acid.[10] ^{19}F spectra were collected with 8192 data points and a spectral width of 20 ppm (acquisition time of 0.18 s) using a recycle delay of 1.5 s with the carrier frequency set to -62.5 ppm. All 1D ^{19}F spectra were processed and analyzed in Topspin (Bruker) and MestreNova. The FIDs were apodized with an exponential window function using a line broadening factor of 20 Hz, zero-filled to 16000 points, and baselines were corrected with a polynomial fit.

2. SUPPLEMENTAL FIGURES

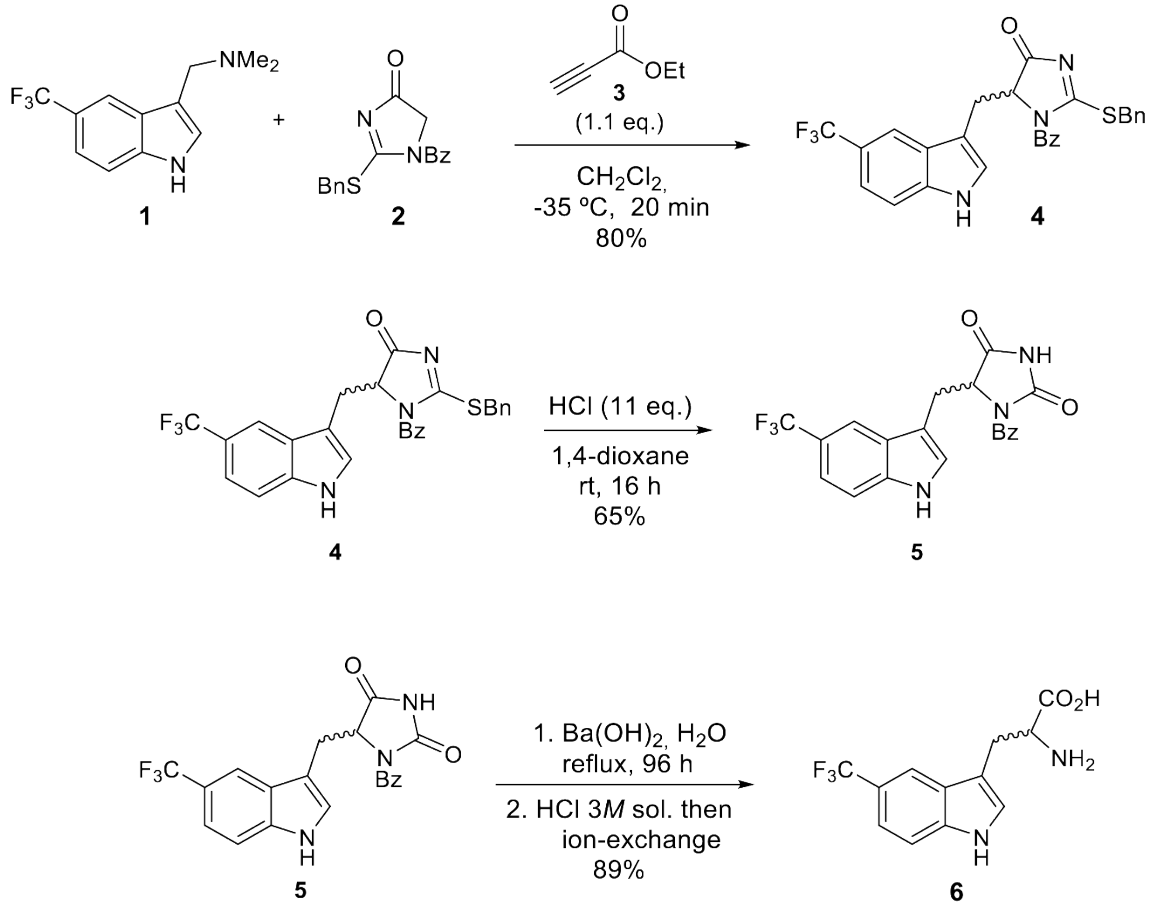

Figure S1. Chemical synthesis of tfmW

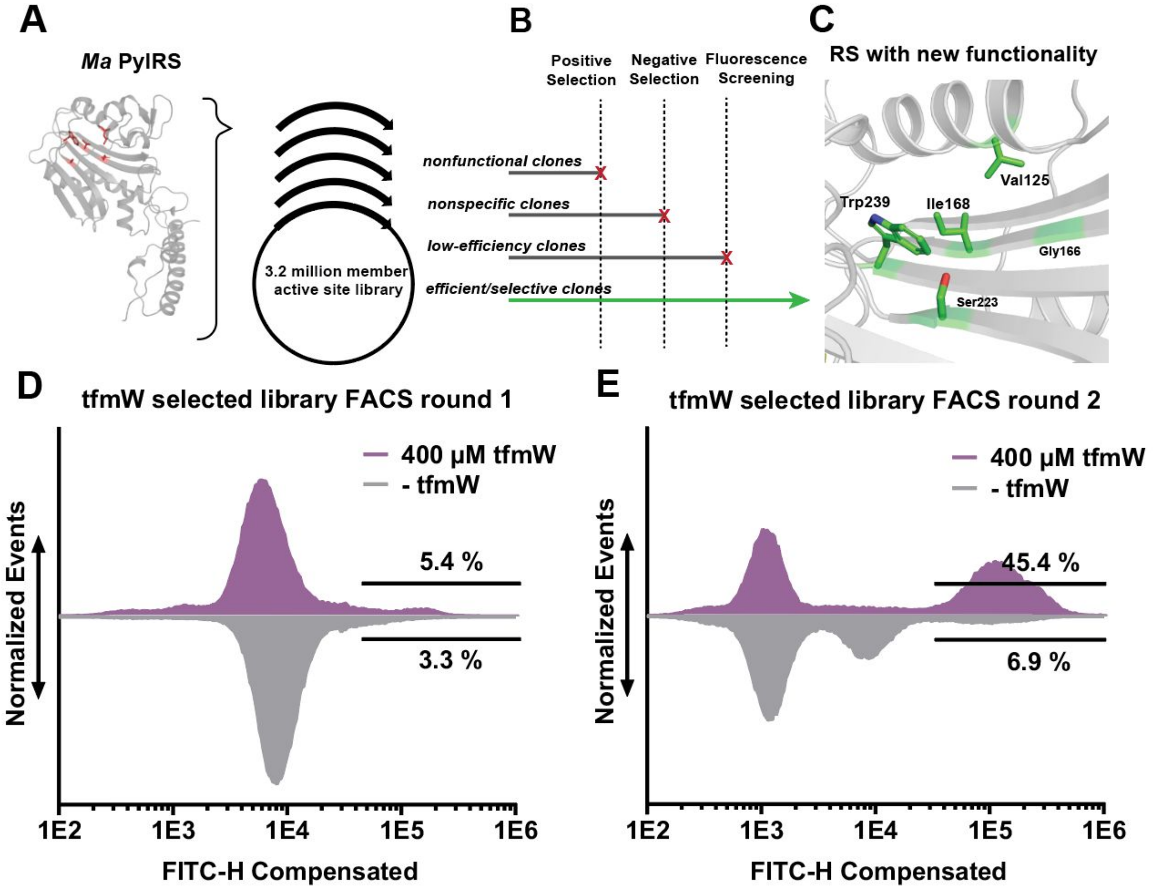

Figure S2. Selection of the tfmW-specific synthetase. (A) a 3.2 million-member *Ma*RS pBK plasmid library with saturation at five active site residues was propagated. (B) The library was culled using positive and negative selections. Fluorescence screening was used to concentrate the library for members with efficient and specific tfmW encoding and to isolate individual clones. (C) Active site of an RS (PDB ID 6JP2), highlighting the changes L125V, N166G, V168I, A223S, with the conserved W239 (orientation changes predicted by Chai-2). (D) Round 1 FACS histograms of cultures expressing the pBK library after negative selection. Cells were grown with 400 µM tfmW, and the most fluorescent cells, at 5.4 %, were passaged for a second round of FACS. In a culture without tfmW, only 3.3 % of cells reached the same fluorescence intensity. (E) Round 2 FACS histograms of cultures expressing the pBK library isolated from Round 1. Of the culture grown with 400 µM tfmW, the largest number of fluorescent cells, at 45.4 %, was collected for isolation. In a culture without ncAA, only 6.9 % of cells reached the same intensity of fluorescence.

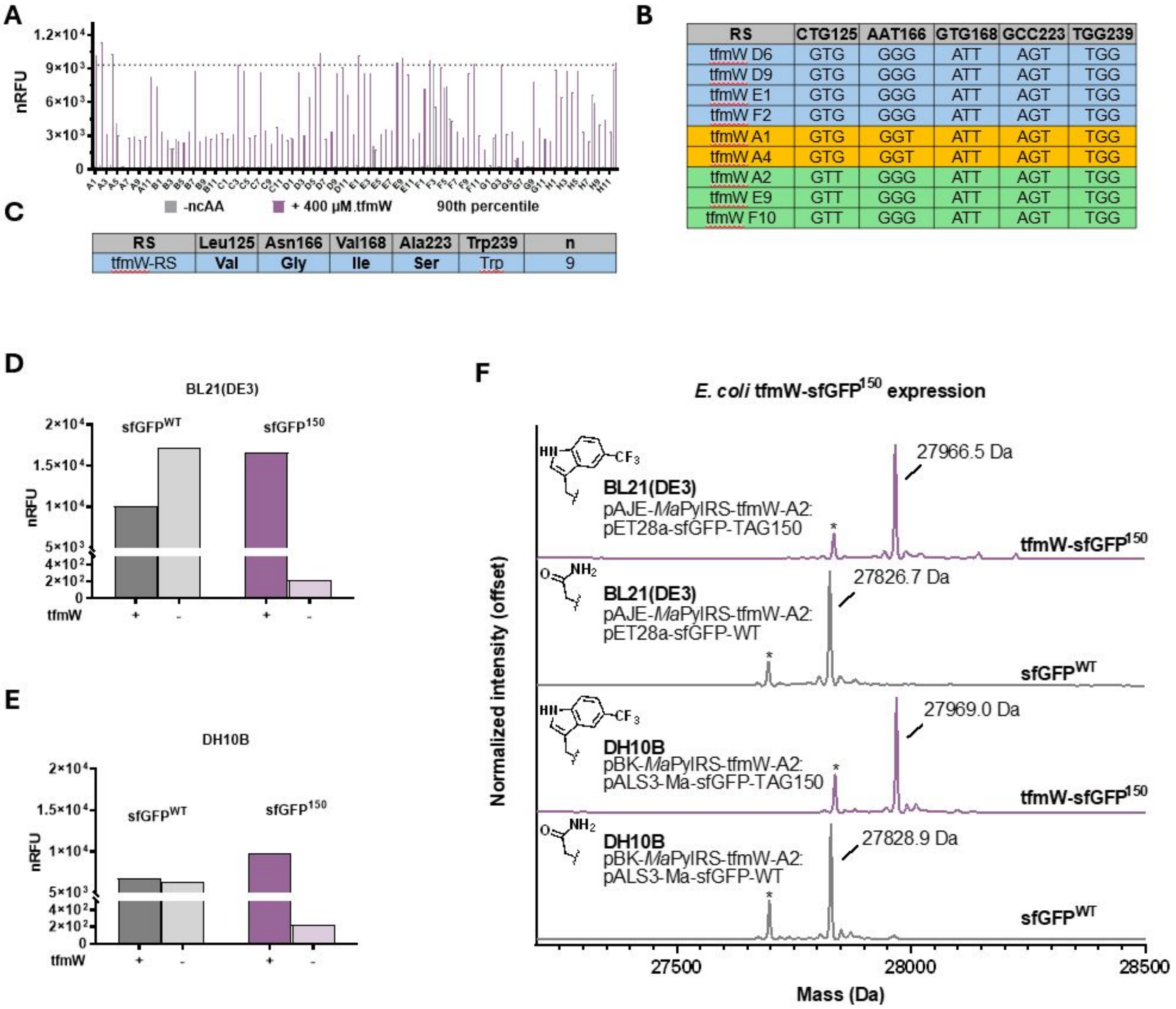

RS	CTG125	AAT166	GTG168	GCC223	TGG239
tfmW D6	GTG	GGG	ATT	AGT	TGG
tfmW D9	GTG	GGG	ATT	AGT	TGG
tfmW E1	GTG	GGG	ATT	AGT	TGG
tfmW F2	GTG	GGG	ATT	AGT	TGG
tfmW A1	GTG	GGT	ATT	AGT	TGG
tfmW A4	GTG	GGT	ATT	AGT	TGG
tfmW A2	GTT	GGG	ATT	AGT	TGG
tfmW E9	GTT	GGG	ATT	AGT	TGG
tfmW F10	GTT	GGG	ATT	AGT	TGG

RS	Leu125	Asn166	Val168	Ala223	Trp239	n
tfmW-RS	Val	Gly	Ile	Ser	Trp	9

Figure S3. Efficiency, fidelity, and expression of tfmW-sfGFP^{150} across *E. coli* expression hosts. (A) Efficiency and fidelity of 95 individual clones after fluorescence screening. Clones were ranked by normalized fluorescence (nRFU), and the nine most fluorescent clones were sequenced (above the dotted line). (B) Sequencing revealed three genetically distinct clones (blue, gold, and green). The site 125 codon varied between GTG and GTT, while the site 166 codon varied between GGG and GGT. Each pair is redundant in translation, resulting in a single amino acid sequence (C). (D-E) sfGFP expression in 50 mL autoinducing medium using (D) DH10B as the expression host or (E) BL21(*DE3*) as the expression host, in the presence of 400 µM tfmW. (F) ESI-Q-TOF mass spectrometry of tfmW-sfGFP^{150} from *E. coli* expression hosts. The observed masses of tfmW-sfGFP^{150} were 27966.5 Da and 27969.0 Da (27967.6 Da predicted) using BL21(*DE3*) and DH10B, respectively, while the observed masses of sfGFP^{wt} were 27826.7 Da and 27828.9 Da (27827.5 Da predicted) using BL21(*DE3*) and DH10B, respectively. All *E. coli* samples showed a small amount of N-terminal fMet cleavage (*) as indicated by peaks at 130-131 Da less than the full protein mass.

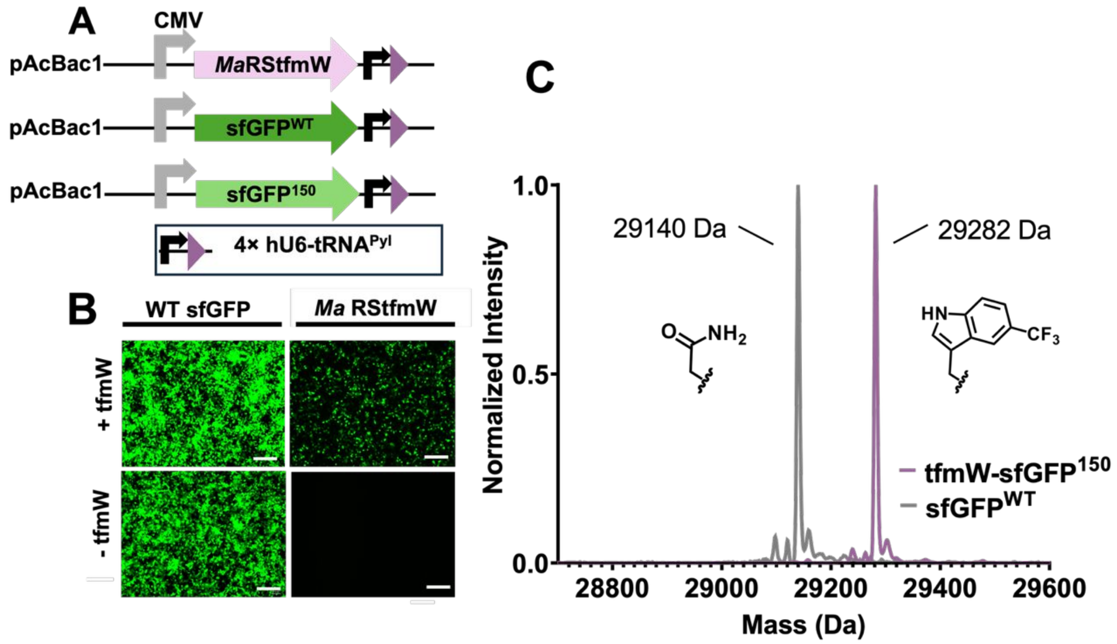

Figure S4: Genetic encoding of tfmW into sfGFP^{150} in HEK293T cells (A) Schematic view of the plasmid construct used for site-specific incorporation of tfmW into sfGFP in HEK293T cells. (B) Fluorescence microscopy images of HEK293T cells expressing sfGFP^{WT} and sfGFP^{150} co-transfected with *Ma* RS tfmWA2/tRNA$_{CUA}$ pair. Cells were cultured in the presence (top) or absence (bottom) of tfmW (100 μM). (C) The observed mass of tfmW-sfGFP^{150} was 29282 Da (29283 Da predicted), and the observed mass for sfGFP^{WT} was 29140 Da (29142 Da predicted).

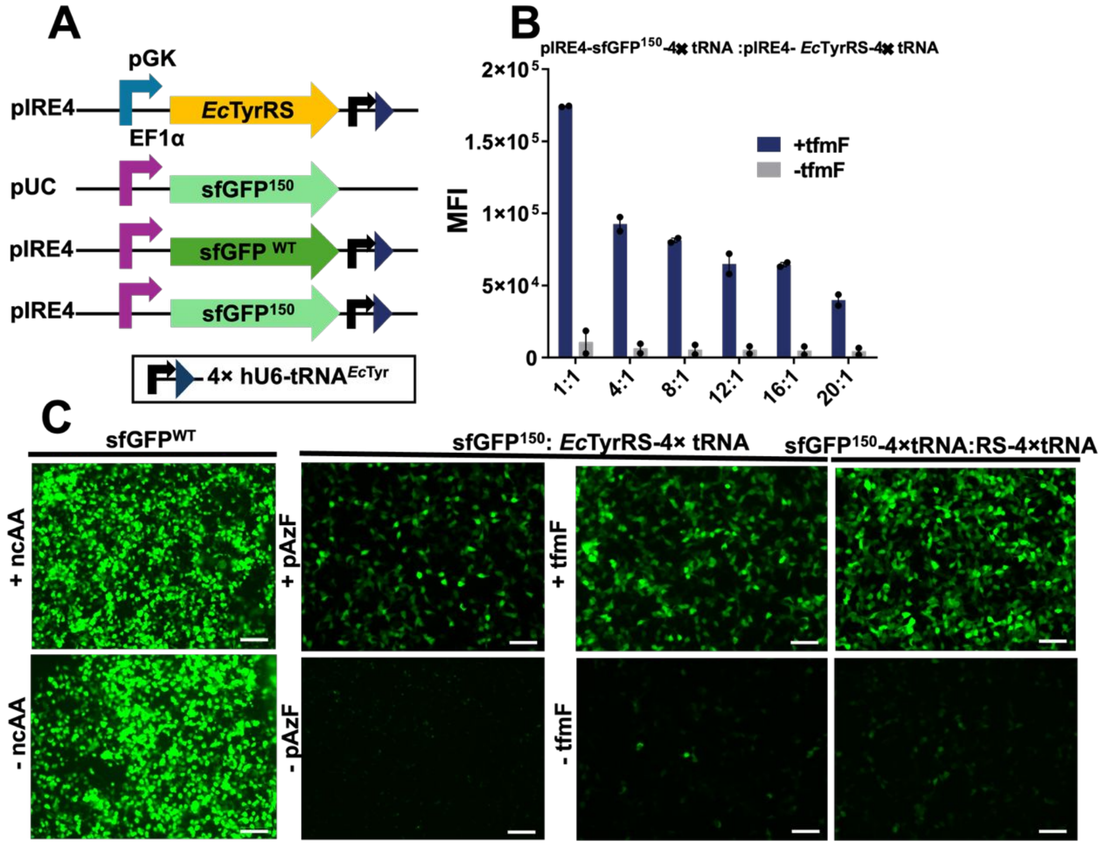

Figure S5: Genetic encoding of tfmF into sfGFP^{150} in HEK293T cells (A) Schematic representation of the plasmid construct used for site-specific incorporation of tfmF into sfGFP^{150} in HEK293T cells. (B) Quantitative analysis of mean fluorescence intensity of sfGFP^{150}expression in the presence of tfmF, as determined by flow cytometry with an increasing ratio of sfGFP^{150TAG}: RS (C) Fluorescence microscopy images of HEK293T cells expressing sfGFP^{WT} and sfGFP^{150} co-transfected with *Ec*TyrRS/tRNA$_{CUA}$ pair. Images in the top and bottom panels show the expression of GFP in the presence of tfmF (500 µM) or in the absence of tfmF. All images were acquired 48 hours post-transfection. Scale bar = 100 µm.

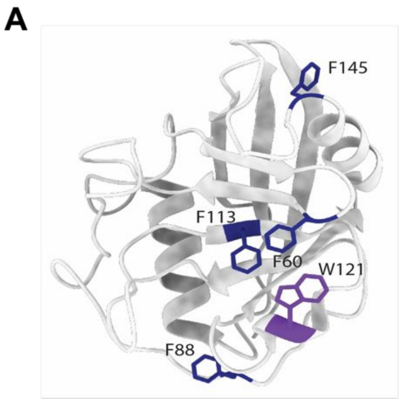

C

CypA variant (HEK293T)	Residue	Yield (mg)	Yield (mg/plate)
tfmW CypA	W121	0.95	0.106
tfmF CypA	F60	0.774	0.097
WT CypA-His$_6$	WT	1.25	0.125

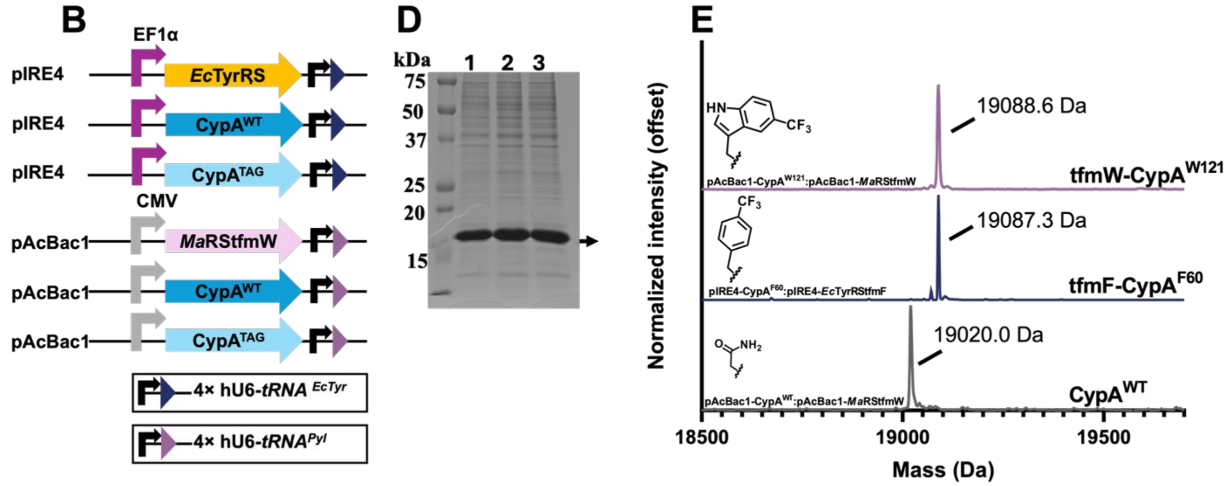

Figure S6: Site-specific introduction of tfm ncAAs into CypA^{60} and CypA^{W121} in HEK293T cells (A) Ribbon representation of the CypA crystal structure (PDB ID: 3K0N), highlighting the five positions (F60, F88, F113, W121, and F145) whose codons were changed into an amber codon (UAG). Changed residues are shown in blue for tfmF and purple for tfmW. (B) Schematic representation of the plasmid constructs used for genetic code expansion for incorporation of tfmF and tfmW into CypA. Key components include the U6 promoter for tRNA expression, the EF1α promoter for consistent expression of CypA^{WT} and CypA^{TAG} in the pIRE4 vector, and the pGK promoter for *Ec*TyrRS, the CMV promoter for the expression of CypA^{WT}, CypA^{TAG,} and *Ma*RS tfmWA2/tRNA$_{CUA}$ in the pAcBac1 vector. (C) Quantification of the final yield of the CypA^{WT} and tfm-CypA^{TAG} variants after purification from HEK293T cells. (D) SDS-PAGE gel of purified proteins isolated from HEK293T cells after transient transfection (E) ESI-MS analysis, confirming the site-specific incorporation of tfmF and tfmW into CypA expressed in HEK293T cells, compared to the CypA^{WT}. The observed masses of tfmW-CypA^{121} and tfmF-CypA^{60} were 19088.6 Da (predicted with N-terminal acetylation 19088.5 Da) and 19087.3 Da (predicted with acetylation 19087.3 Da), respectively. The experimental mass for CypA^{WT} was 19020.2 Da (predicted with acetylation 19020.4 Da).

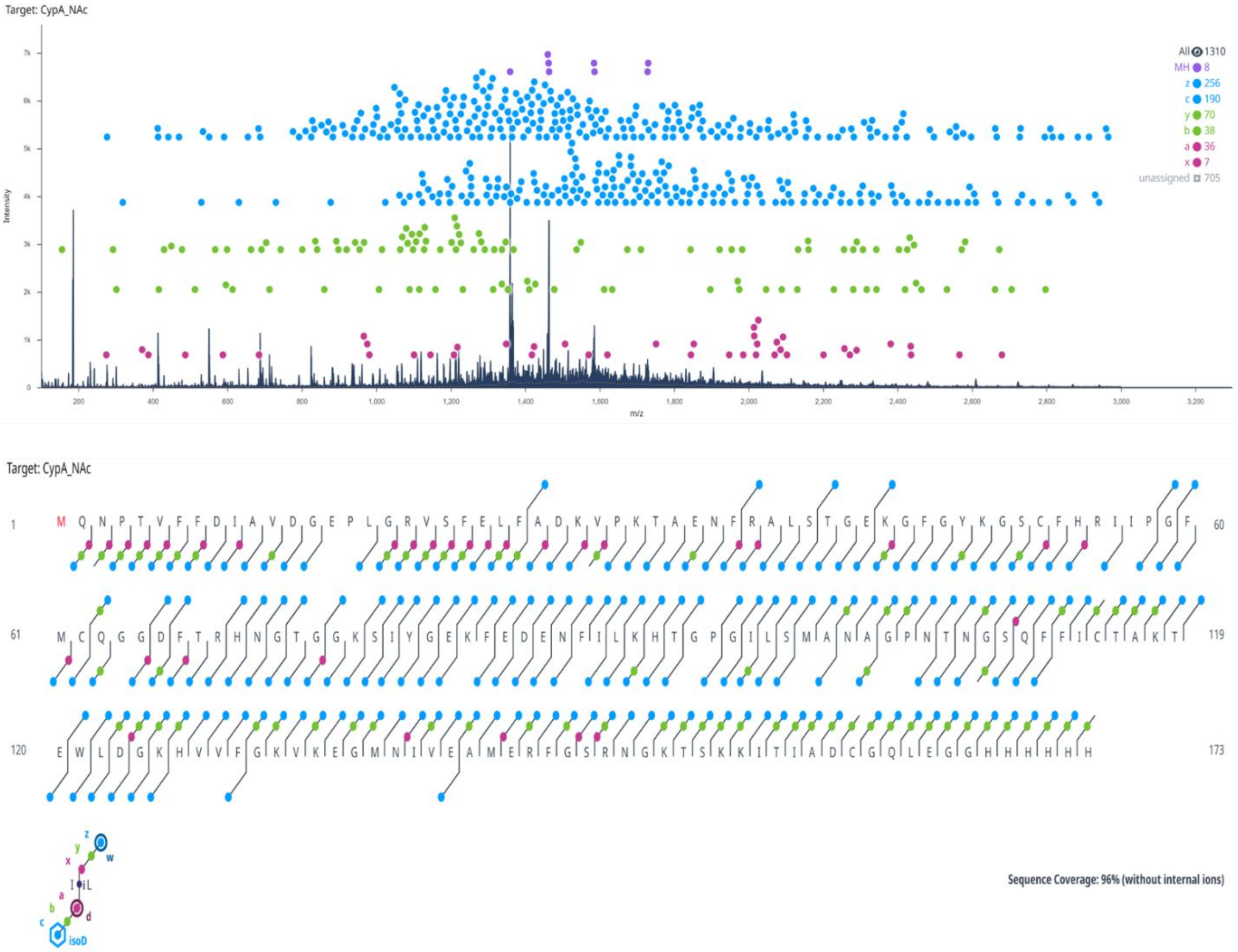

Figure S7: Top-down mass spectrometry (MS) spectrum (top) and sequence coverage (bottom) showing N-terminal acetylation of $CypA^{WT}$

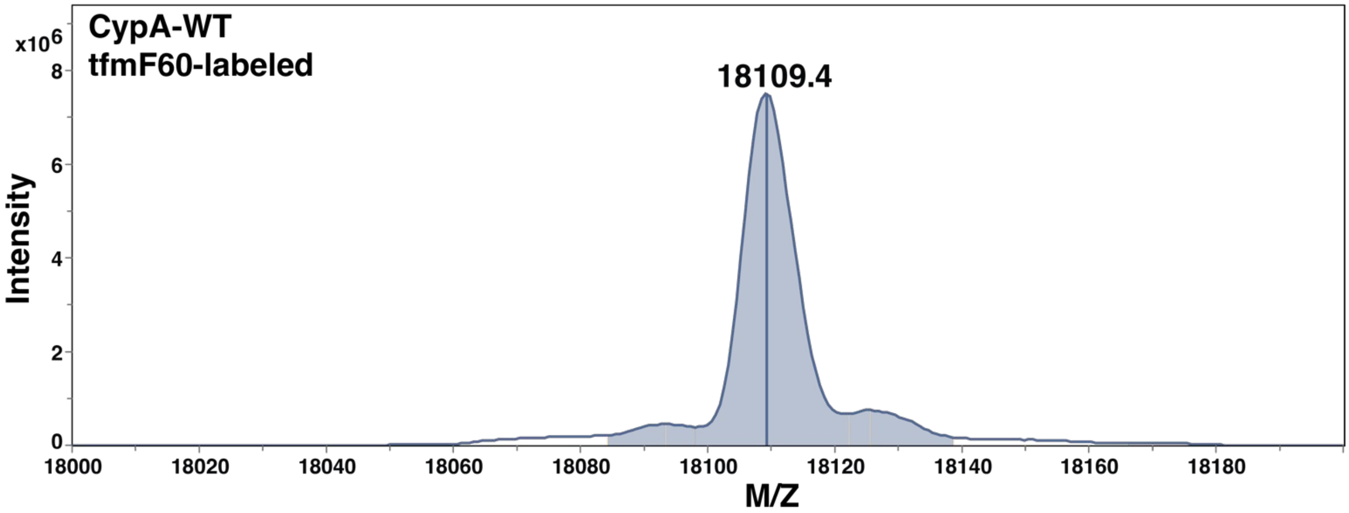

Figure S8: Mass spectrum of tfmF-CypA^{F60} purified from *E. coli*. The observed and predicted molecular masses are 18109.4 and18109, respectively.

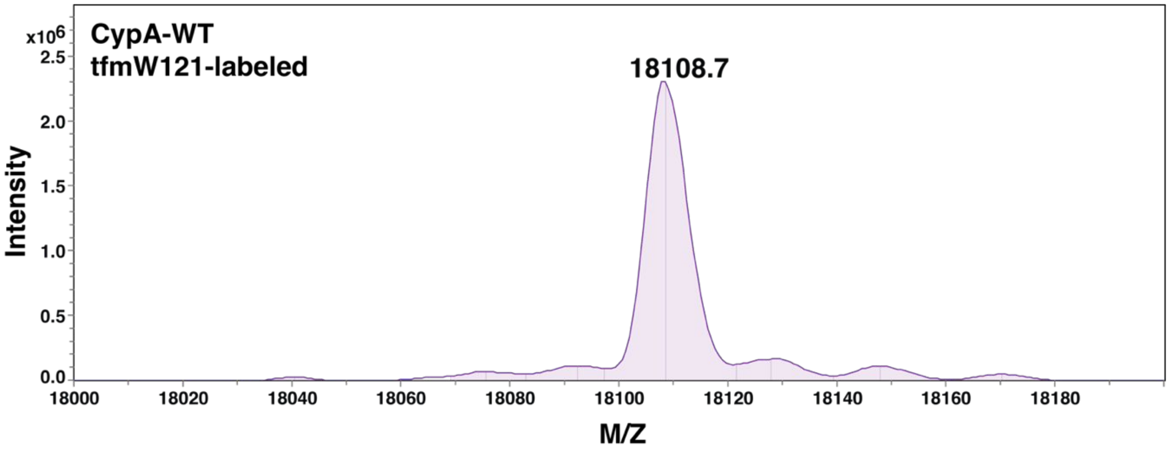

Figure S9: Mass spectrum of tfmW-CypA^{W121} purified from *E. coli*. The observed and predicted molecular masses are 18108.7 and 18109, respectively.

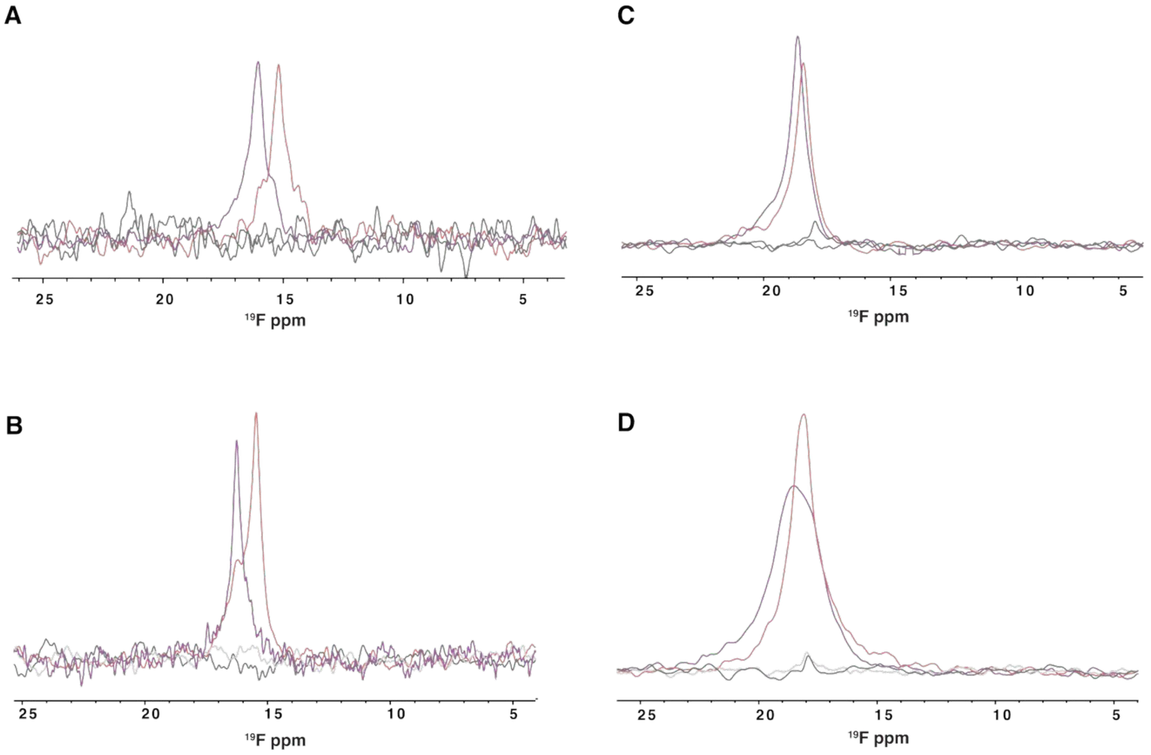

Figure S10: Full spectral window of the in-cell ^{19}F NMR spectra of tfmF- and tfmW-labeled CypA. Spectra of tfmF-CypA^{60} without and with CsA are shown in blue and pink, respectively, in cells after electroporation (A) or following transient transfection (B). Spectra of tfmW-CypA^{121} without and with CsA are shown in purple and pink, respectively, after electroporation (C), and following transient transfection (D). Supernatant control spectra, recorded after the in-cell spectra, are shown in gray.

SUPPLEMENTAL TABLES

Table 1: List of primers used for cloning $sfGFP^{WT}$, $sfGFP^{150TAG}$, $CypA^{WT}$, and $CypA^{TAG}$ in pUC, pIRE4, pET41, and pAcBac1 vectors

Name	***Sequence***
pAcBac-CypA-FLAG-Rev	acgcgttgaattcattacttatcgtcgtcatccttgtaatcacctccttcgagttgtccac
pAcBac-CypA-His-Rev	cgttgaattcattagtggtgatggtggtgatgacctccttcgagttgtcc
pAcBac-NheI-CypA-FWD	ccaagctggctagcgtttaaacttaagcttgccaccatgcagaaccccaccgtgttc
Amp_CypA-for-pIRE4-FWD	cctgctagcgtttaaacttaagcttgccaccatgcagaaccccaccgtgttcttcg
Amp_CypA-for-pIRE4-REV	gcggccgccactgtgctggatatctgcagaattcattattcgagttgtccacagtcagca
Ef1a-EF1aCypA-for-pIRE4-FWD	cctgattctgtggataaccgtattaccgccatgcacgcgttgctccggtgcccgtcagtg
EF1a-sfGFP-for-pIRE4-Rev	gcggccgccactgtgctggatatctgcagaattcattagtggtgatggtggtgatgagtagaatc
pIRE4-CYP-A-Nhe1-FWD	ggtgtcgtgatggctagcgtttaaac
pIRE4-CypA-C-His-REV	atatctgcagaattcattacttatcgtcgtcatccttgtaatcacctccttcgagttgtccacagtcagc
pET41-CypA-F60TAG-Rev	ccagctactcggtcttcgcgg
pET41-CypA-W121TAG-Rev	gcacatctaacccggaatgatacggtgaa

Table 2: Description of the plasmids used for tfmF and tfmW incorporation in HEK293T cells

**Plasmid name**	**Gene of Interest**	**Promoter**	**tRNA**	**Purification tags**	**Antibiotic resistance**
pU-CypA^{WT}	CypA^{WT}	*EF-1α*	No	No	Neo/Kan
pUC-P2-CypA^{F60TAG}	CypA^{F60TAG}	*EF-1α*	No	No	Neo/Kan
pUC-P2-CypA^{F88TAG}	CypA^{F88TAG}	*EF-1α*	No	No	Neo/Kan
pUC-P2-CypA^{F113TAG}	CypA^{F113TAG}	*EF-1α*	No	No	Neo/Kan
pUC-P2-CypA^{W121TAG}	CypA^{W121TAG}	*EF-1α*	No	No	Neo/Kan
pUC-P2-CypA^{F145TAG}	CypA^{F145TAG}	*EF-1α*	No	No	Neo/Kan
pIRE4-CypA^{WT}	CypA^{WT}	*EF-1α*	4-tRNA^{Ectyr}	No	Neo/Kan
pIRE4-CypA^{F60TAG}	CypA^{F60TAG}	*EF-1α*	4-tRNA^{Ectyr}	No	Neo/Kan
pIRE4-CypA^{F88TAG}	CypA^{F88TAG}	*EF-1α*	4-tRNA^{Ectyr}	No	Neo/Kan
pIRE4-CypA^{F113TAG}	CypA^{F113TAG}	*EF-1α*	4-tRNA^{Ectyr}	No	Neo/Kan
pIRE4-CypA^{W121TAG}	CypA^{W121TAG}	*EF-1α*	4-tRNA^{Ectyr}	No	Neo/Kan
pIRE4-CypA^{F145TAG}	CypA^{F145TAG}	*EF-1α*	4-tRNA^{Ectyr}	No	Neo/Kan
pGA2-GK-Ec-OMeYRS-4xtRNA	*EcYRS*	*PGK*	4-tRNA^{Ectyr}	No	Neo/Kan
pIRE4-CypA^{WT}-FLAG	CypA^{WT}	*EF-1α*	4-tRNA	C-terminal FLAG	Neo/Kan
pIRE4-CypA^{F60TAG}-FLAG	CypA^{F60TAG}	*EF-1α*	4-tRNA^{Ectyr}	C-terminal FLAG	Neo/Kan
pIRE4-CypA^{F88TAG}-FLAG	CypA^{F88TAG}	*EF-1α*	4-tRNA^{Ectyr}	C-terminal FLAG	Neo/Kan
pIRE4-CypA^{F113TAG}-FLAG	CypA^{F113TAG}	*EF-1α*	4-tRNA^{Ectyr}	C-terminal FLAG	Neo/Kan
pIRE4-CypA^{W12TAG1}-FLAG	CypA^{W121TAG}	*EF-1α*	4-tRNA^{Ectyr}	C-terminal FLAG	Neo/Kan
pIRE4-CypA^{F14TAG}-FLAG	CypA^{F145TAG}	*EF-1α*	4-tRNA^{Ectyr}	C-terminal FLAG	Neo/Kan
pIRE4-CypA^{WT}-His$_6$	CypA^{WT}	*EF-1α*	4-tRNA^{Ectyr}	C-terminal His$_6$	Neo/Kan
pIRE4-CypA^{F60TAG}-His$_6$	*CypA^{F60TAG}*	*EF-1α*	4-tRNA^{Ectyr}	C-terminal His$_6$	Neo/Kan
pAcBac1-CypA-^{WT}-FLAG	CypA^{WT}	*CMV*	4-tRNA^{Pyl}	C-terminal FLAG	Amp
pAcBac1-CypA^{W121TAG}-FLAG	CypA^{W121TAG}	*CMV*	4-tRNA^{Pyl}	C-terminal FLAG	Amp
pAcBac1-CypA^{WT}-His$_6$	CypA^{WT}	*CMV*	4-tRNA^{Pyl}	C-terminal His$_6$	Amp
pAcBac1-CypA^{W12TAG}-His$_6$	CypA^{W121TAG}	*CMV*	4-tRNA^{Pyl}	C-terminal His$_6$	Amp
pAcBAc1-MaRS-tfmW(A2)	*Ma*RStfmW(A2)	*CMV*	4-tRNA^{Pyl}	C-terminal His$_6$	Amp
pUC-P2-sfGFP^{WT}	sfGFP^{WT}	*EF-1α*	*No tRNA*	No	Neo/Kan
pUC-P2-sfGFP^{TAG}	sfGFP^{TAG}	*EF-1α*	No tRNA	No	Neo/Kan
pGA1-EF1α–sfGFP^{WT}-His$_6$	sfGFP^{WT}	*EF-1α*	4-tRNA^{Ectyr}	C-terminal His$_6$	Neo/Kan
pGA1-EF1α–sfGFP^{150TAG}-4xtRNA	sfGFP^{150TAG}	*EF-1α*	4-tRNA^{Ectyr}	C-terminal His$_6$	Neo/Kan
pAcBac1-sfGFP^{WT}-His$_6$	sfGFP^{WT}	*CMV*	4-tRNA^{Pyl}	C-terminal His$_6$	Amp
pAcBac1-sfGFP^{150TAG}-His$_6$	sfGFP^{150TAG}	*CMV*	4-tRNA^{Pyl}	C-terminal His$_6$	Amp

Table 3: Sequence of CypA^{WT} and CypA^{TAG} inserts used for tfmF and tfmW incorporation.

**Name**	**Sequence**
CypA^{WT}	ATGCAGAACCCCACCGTGTTCTTCGACATTGCCGTCGACGGCGAGCCCTTGGGCCGCGTCTCCTTTGAGC<br>TGTTTGCAGACAAGGTCCCAAAGACAGCAGAAAATTTTCGTGCTCTGAGCACTGGAGAGAAAGGATTTG<br>GTTATAAGGGTTCCTGCTTTCACAGAATTATTCCAGGGTTTATGTGTCAGGGTGGTGACTTCACACGCCA<br>TAATGGCACTGGTGGCAAGTCCATCTATGGGGAGAAATTTGAAGATGAGAACTTCATCCTAAAGCATAC<br>GGGTCCTGGCATCTTGTCCATGGCAAATGCTGGACCCAACACAAATGGTTCCCAGTTTTTCATCTGCACT<br>GCCAAGACTGAGTGGTTGGATGGCAAGCATGTGGTGTTTGGCAAAGTGAAAGAAGGCATGAATATTGTG<br>GAGGCCATGGAGCGCTTTGGGTCCAGGAATGGCAAGACCAGCAAGAAGATCACCATTGCTGACTGTGGA<br>CAACTCGAATAA
CypA^{F60TAG}	ATGCAGAACCCCACCGTGTTCTTCGACATTGCCGTCGACGGCGAGCCCTTGGGCCGCGTCTCCTTTGAGC<br>TGTTTGCAGACAAGGTCCCAAAGACAGCAGAAAATTTTCGTGCTCTGAGCACTGGAGAGAAAGGATTTG<br>GTTATAAGGGTTCCTGCTTTCACAGAATTATTCCAGGG**TAG**ATGTGTCAGGGTGGTGACTTCACACGCCA<br>TAATGGCACTGGTGGCAAGTCCATCTATGGGGAGAAATTTGAAGATGAGAACTTCATCCTAAAGCATAC<br>GGGTCCTGGCATCTTGTCCATGGCAAATGCTGGACCCAACACAAATGGTTCCCAGTTTTTCATCTGCACT<br>GCCAAGACTGAGTGGTTGGATGGCAAGCATGTGGTGTTTGGCAAAGTGAAAGAAGGCATGAATATTGTG<br>GAGGCCATGGAGCGCTTTGGGTCCAGGAATGGCAAGACCAGCAAGAAGATCACCATTGCTGACTGTGGA<br>CAACTCGAATAA
CypA^{F88TAG}	ATGCAGAACCCCACCGTGTTCTTCGACATTGCCGTCGACGGCGAGCCCTTGGGCCGCGTCTCCTTTGAGC<br>TGTTTGCAGACAAGGTCCCAAAGACAGCAGAAAATTTTCGTGCTCTGAGCACTGGAGAGAAAGGATTTG<br>GTTATAAGGGTTCCTGCTTTCACAGAATTATTCCAGGGTTTATGTGTCAGGGTGGTGACTTCACACGCCA<br>TAATGGCACTGGTGGCAAGTCCATCTATGGGGAGAAATTTGAAGATGAGAAC**TAG**ATCCTAAAGCATAC<br>GGGTCCTGGCATCTTGTCCATGGCAAATGCTGGACCCAACACAAATGGTTCCCAGTTTTTCATCTGCACT<br>GCCAAGACTGAGTGGTTGGATGGCAAGCATGTGGTGTTTGGCAAAGTGAAAGAAGGCATGAATATTGTG<br>GAGGCCATGGAGCGCTTTGGGTCCAGGAATGGCAAGACCAGCAAGAAGATCACCATTGCTGACTGTGGA<br>CAACTCGAATAA
CypA^{F113TAG}	ATGCAGAACCCCACCGTGTTCTTCGACATTGCCGTCGACGGCGAGCCCTTGGGCCGCGTCTCCTTTGAGC<br>TGTTTGCAGACAAGGTCCCAAAGACAGCAGAAAATTTTCGTGCTCTGAGCACTGGAGAGAAAGGATTTG<br>GTTATAAGGGTTCCTGCTTTCACAGAATTATTCCAGGGTTTATGTGTCAGGGTGGTGACTTCACACGCCA<br>TAATGGCACTGGTGGCAAGTCCATCTATGGGGAGAAATTTGAAGATGAGAACTTCATCCTAAAGCATAC<br>GGGTCCTGGCATCTTGTCCATGGCAAATGCTGGACCCAACACAAATGGTTCCCAGTTT**TAG**ATCTGCACT<br>GCCAAGACTGAGTGGTTGGATGGCAAGCATGTGGTGTTTGGCAAAGTGAAAGAAGGCATGAATATTGTG<br>GAGGCCATGGAGCGCTTTGGGTCCAGGAATGGCAAGACCAGCAAGAAGATCACCATTGCTGACTGTGGA<br>CAACTCGAATAA
CypA^{F145TAG}	ATGCAGAACCCCACCGTGTTCTTCGACATTGCCGTCGACGGCGAGCCCTTGGGCCGCGTCTCCTTTGAGC<br>TGTTTGCAGACAAGGTCCCAAAGACAGCAGAAAATTTTCGTGCTCTGAGCACTGGAGAGAAAGGATTTG<br>GTTATAAGGGTTCCTGCTTTCACAGAATTATTCCAGGGTTTATGTGTCAGGGTGGTGACTTCACACGCCA<br>TAATGGCACTGGTGGCAAGTCCATCTATGGGGAGAAATTTGAAGATGAGAACTTCATCCTAAAGCATAC<br>GGGTCCTGGCATCTTGTCCATGGCAAATGCTGGACCCAACACAAATGGTTCCCAGTTTTTCATCTGCACT<br>GCCAAGACTGAGTGGTTGGATGGCAAGCATGTGGTGTTTGGCAAAGTGAAAGAAGGCATGAATATTGTG<br>GAGGCCATGGAGCGC**TAG**GGGTCCAGGAATGGCAAGACCAGCAAGAAGATCACCATTGCTGACTGTGG<br>ACAACTCGAATAA
CypA^{W121TAG}	ATGCAGAACCCCACCGTGTTCTTCGACATTGCCGTCGACGGCGAGCCCTTGGGCCGCGTCTCCTTTGAGC<br>TGTTTGCAGACAAGGTCCCAAAGACAGCAGAAAATTTTCGTGCTCTGAGCACTGGAGAGAAAGGATTTG<br>GTTATAAGGGTTCCTGCTTTCACAGAATTATTCCAGGGTTTATGTGTCAGGGTGGTGACTTCACACGCCA<br>TAATGGCACTGGTGGCAAGTCCATCTATGGGGAGAAATTTGAAGATGAGAACTTCATCCTAAAGCATAC<br>GGGTCCTGGCATCTTGTCCATGGCAAATGCTGGACCCAACACAAATGGTTCCCAGTTTTTCATCTGCACT<br>GCCAAGACTGAG**TAG**TTGGATGGCAAGCATGTGGTGTTTGGCAAAGTGAAAGAAGGCATGAATATTGTG<br>GAGGCCATGGAGCGCTTTGGGTCCAGGAATGGCAAGACCAGCAAGAAGATCACCATTGCTGACTGTGGA<br>CAACTCGAATAA
CypA^{WT} (E. coli optimized for expression)	ATGCAGAACCCCACCGTGTTCTTCGACATTGCCGTCGACGGCGAGCCCTTGGGCCGCGTCTCCTTTGAGC<br>TGTTTGCAGACAAGGTCCCAAAGACAGCAGAAAATTTTCGTGCTCTGAGCACTGGAGAGAAAGGATTTG<br>GTTATAAGGGTTCCTGCTTTCACAGAATTATTCCAGGGTTTATGTGTCAGGGTGGTGACTTCACACGCCA<br>TAATGGCACTGGTGGCAAGTCCATCTATGGGGAGAAATTTGAAGATGAGAACTTCATCCTAAAGCATAC<br>GGGTCCTGGCATCTTGTCCATGGCAAATGCTGGACCCAACACAAATGGTTCCCAGTTTTTCATCTGCACT<br>GCCAAGACTGAGTGGTTGGATGGCAAGCATGTGGTGTTTGGCAAAGTGAAAGAAGGCATGAATATTGTG<br>GAGGCCATGGAGCGCTTTGGGTCCAGGAATGGCAAGACCAGCAAGAAGATCACCATTGCTGACTGTGGA<br>CAACTCGAATAA

Table 4: Description of the plasmids used for tfmF and tfmW incorporation in *E.coli*

Plasmid name	Gene of Interest	Purification tags	Antibiotic resistance
pet41-CypA^{WT}	CypA^{WT}	N-terminal His_6	Kanamycin
pet41-CypA^{F60TAG}	CypA^{F60TAG}	N-terminal His_6	Kanamycin
pet41-CypA^{F88TAG}	CypA^{F88TAG}	N-terminal His_6	Kanamycin
pet41-CypA^{F113TAG}	CypA^{F113TAG}	N-terminal His_6	Kanamycin
pet41-CypA^{W121TAG}	CypA^{W121TAG}	N-terminal His_6	Kanamycin
pet41-CypA^{F145TAG}	CypA^{F145TAG}	N-terminal His_6	Kanamycin
pAJE90-Ma-tfmW-RSA2	tfmW-RSA2	None	Spectinomycin
pDule2-tfmF-RS	tfmF-RS	None	Streptomycin

Table 5: Summary of purified protein yields of tfmF and tfmW labeled proteins purified from *E. coli*

Protein (variant)	Residue	Label	Growth media	Yield (mg/L)
sfGFP^{WT}	N150	None	ZY	260
sfGFP^{N150TAG}	N150	tfmF	ZY	260
sfGFP^{N150TAG}	N150	tfmW	LB	223
CypA^{WT}	F60	None	LB	35
CypA^{F60TAG}	F60	tfmF	LB	20
CypA^{WT}	F60	None	M9	20
CypA^{F60TAG}	F60	tfmF	M9	25
CypA^{W121TAG}	W121	tfmW	M9	20

3. REFERENCES

(1) Hernández, I.; Domínguez, G.; Soloshonok, V. A.; Landa, A.; Oiarbide, M. Ynone Promoted Deaminative Coupling of Gramines with C- and N-Nucleophiles. *J Org Chem* **2024**, *89* (23), 17291-17309. DOI: 10.1021/acs.joc.4c01895 From NLM.
(2) Villaescusa, L.; Hernández, I.; Azcune, L.; Rudi, A.; Mercero, J. M.; Landa, A.; Oiarbide, M.; Palomo, C. Rigidified Bis(sulfonyl)ethylenes as Effective Michael Acceptors for Asymmetric Catalysis: Application to the Enantioselective Synthesis of Quaternary Hydantoins. *The Journal of Organic Chemistry* **2023**, *88* (2), 972-987. DOI: 10.1021/acs.joc.2c02403.
(3) Alexander, N. D.; Gangarde, Y. M.; Bednar, R. M.; Karplus, P. A.; Cooley, R. B.; Mehl, R. A. Selecting aminoacyl-tRNA synthetase/tRNA pairs for efficient genetic encoding of noncanonical amino acids into proteins. *Nat Protoc* **2025**. DOI: 10.1038/s41596-025-01241-w.
(4) Avila-Crump, S.; Hemshorn, M. L.; Jones, C. M.; Mbengi, L.; Meyer, K.; Griffis, J. A.; Jana, S.; Petrina, G. E.; Pagar, V. V.; Karplus, P. A.; et al. Generating Efficient Methanomethylophilus alvus Pyrrolysyl-tRNA Synthetases for Structurally Diverse Non-Canonical Amino Acids. *ACS Chem Biol* **2022**, *17* (12), 3458-3469. DOI: 10.1021/acschembio.2c00639.
(5) Jana, S.; Evans, E. G. B.; Jang, H. S.; Zhang, S.; Zhang, H.; Rajca, A.; Gordon, S. E.; Zagotta, W. N.; Stoll, S.; Mehl, R. A. Ultrafast Bioorthogonal Spin-Labeling and Distance Measurements in Mammalian Cells Using Small, Genetically Encoded Tetrazine Amino Acids. *J Am Chem Soc* **2023**, *145* (27), 14608-14620. DOI: 10.1021/jacs.3c00967.

(6) Seidel, L.; Zarzycka, B.; Zaidi, S. A.; Katritch, V.; Coin, I. Structural insight into the activation of a class B G-protein-coupled receptor by peptide hormones in live human cells. *Elife* **2017**, *6*. DOI: 10.7554/eLife.27711.
(7) Zhang, Y.; Werling, U.; Edelmann, W. SLiCE: a novel bacterial cell extract-based DNA cloning method. *Nucleic Acids Res* **2012**, *40* (8), e55. DOI: 10.1093/nar/gkr1288.
(8) Abshiru, N.; Caron-Lizotte, O.; Rajan, R. E.; Jamai, A.; Pomies, C.; Verreault, A.; Thibault, P. Discovery of protein acetylation patterns by deconvolution of peptide isomer mass spectra. *Nat Commun* **2015**, *6*, 8648. DOI: 10.1038/ncomms9648.
(9) Jackson, J. C.; Hammill, J. T.; Mehl, R. A. Site-specific incorporation of a (19)F-amino acid into proteins as an NMR probe for characterizing protein structure and reactivity. *J Am Chem Soc* **2007**, *129* (5), 1160-1166. DOI: 10.1021/ja064661t.
(10) Lu, M.; Ishima, R.; Polenova, T.; Gronenborn, A. M. (19)F NMR relaxation studies of fluorosubstituted tryptophans. *J Biomol NMR* **2019**, *73* (8-9), 401-409. DOI: 10.1007/s10858-019-00268-y.
